# Risk and protective factors for new‐onset binge eating, low weight, and self‐harm symptoms in >35,000 individuals in the UK during the COVID‐19 pandemic

**DOI:** 10.1002/eat.23834

**Published:** 2022-10-31

**Authors:** Helena L. Davies, Christopher Hübel, Moritz Herle, Saakshi Kakar, Jessica Mundy, Alicia J. Peel, Abigail R. ter Kuile, Johan Zvrskovec, Dina Monssen, Kai Xiang Lim, Molly R. Davies, Alish B. Palmos, Yuhao Lin, Gursharan Kalsi, Henry C. Rogers, Shannon Bristow, Kiran Glen, Chelsea Mika Malouf, Emily J. Kelly, Kirstin L. Purves, Katherine S. Young, Matthew Hotopf, Cherie Armour, Andrew M. McIntosh, Thalia C. Eley, Janet Treasure, Gerome Breen

**Affiliations:** ^1^ Social, Genetic and Developmental Psychiatry Centre, Institute of Psychiatry, Psychology and Neuroscience King's College London London UK; ^2^ UK National Institute for Health and Care Research (NIHR) Biomedical Research Centre South London and Maudsley Hospital London UK; ^3^ Department of Biostatistics and Health Informatics Institute of Psychiatry, Psychology and Neuroscience, King's College London London UK; ^4^ Department of Psychological Medicine Institute of Psychiatry, Psychology and Neuroscience, King's College London London UK; ^5^ Research Centre for Stress, Trauma and Related Conditions (STARC), School of Psychology Queen's University Belfast Belfast UK; ^6^ Division of Psychiatry, Centre for Clinical Brain Sciences University of Edinburgh Edinburgh UK; ^7^ Section of Eating Disorders, Department of Psychological Medicine Institute of Psychiatry, Psychology and Neuroscience, King's College London London UK

**Keywords:** comorbidity, eating disorders, mental health, psychiatric disorders, suicidal ideation

## Abstract

**Objective:**

The disruption caused by the COVID‐19 pandemic has been associated with poor mental health, including increases in eating disorders and self‐harm symptoms. We investigated risk and protective factors for the new onset of these symptoms during the pandemic.

**Method:**

Data were from the COVID‐19 Psychiatry and Neurological Genetics study and the Repeated Assessment of Mental health in Pandemics Study (*n* = 36,715). Exposures were socio‐demographic characteristics, lifetime psychiatric disorder, and COVID‐related variables, including SARS‐CoV‐2 infection/illness with COVID‐19. We identified four subsamples of participants without pre‐pandemic experience of our outcomes: binge eating (*n* = 24,211), low weight (*n* = 24,364), suicidal and/or self‐harm ideation (*n* = 18,040), and self‐harm (*n* = 29,948). Participants reported on our outcomes at frequent intervals (fortnightly to monthly). We fitted multiple logistic regression models to identify factors associated with the new onset of our outcomes.

**Results:**

Within each subsample, new onset was reported by: 21% for binge eating, 10.8% for low weight, 23.5% for suicidal and/or self‐harm ideation, and 3.5% for self‐harm. Shared risk factors included having a lifetime psychiatric disorder, not being in paid employment, higher pandemic worry scores, and being racially minoritized. Conversely, infection with SARS‐CoV‐2/illness with COVID‐19 was linked to lower odds of binge eating, low weight, and suicidal and/or self‐harm ideation.

**Discussion:**

Overall, we detected shared risk factors that may drive the comorbidity between eating disorders and self‐harm. Subgroups of individuals with these risk factors may require more frequent monitoring during future pandemics.

**Public Significance:**

In a sample of 35,000 UK residents, people who had a psychiatric disorder, identified as being part of a racially minoritized group, were not in paid employment, or were more worried about the pandemic were more likely to experience binge eating, low weight, suicidal and/or self‐harm ideation, and self‐harm for the first time during the pandemic. People with these risk factors may need particular attention during future pandemics to enable early identification of new psychiatric symptoms.

## 1 INTRODUCTION

During the COVID‐19 pandemic, people living in the UK, as in many other countries, were ordered to stay at home and apply limits to social contact during multiple national and local lockdowns. In its early stages, researchers highlighted that the environment generated by the pandemic was likely to exacerbate eating disorders (Rodgers et al., 2020). In January 2021, the UK's largest eating disorder charity, Beat, reported a 173% increase in demand for their helpline services in comparison with February 2020 (Women and Equalities Committee, 2021). The picture is less clear for self‐harm, with some papers reporting a decrease (Hawton, Casey, et al., 2021; Kapur et al., 2021) and others an increase (Henry et al., 2021) during the pandemic.

Self‐harm and eating disorders are both body‐focussed illnesses (Claes & Muehlenkamp, 2014), argued to be disorders of emotion regulation (Gratz & Roemer, 2008; Haynos & Fruzzetti, 2011) and forms of self‐punishment (Svirko & Hawton, 2007). Reports indicate that up to 72% of people with an eating disorder self‐harm without suicidal intent, and 25%–54% of people who self‐harm report co‐occurrent eating disorder symptoms (Claes & Muehlenkamp, 2014). Similarly, having eating disorder symptoms has been associated with a minimum two‐fold increased likelihood of reporting self‐harm ideation (Wright et al., 2009) and approximately one‐quarter of people with an eating disorder report experiencing suicidal ideation while unwell (Carano et al., 2012; Favaro & Santonastaso, 1997; Milos et al., 2004). Shared risk factors may drive their comorbidity, for example, personality traits, such as negative urgency (Cassin & von Ranson, 2005; Claes et al., 2005; Claes & Muehlenkamp, 2013; Dir et al., 2013; Glenn & Klonsky, 2010; Glenn & Klonsky, 2011).

Compared to before the COVID‐19 pandemic, higher instances of binge eating have been reported during the pandemic by both individuals in the general population (Phillipou et al., 2020) and patients with a history of binge‐eating disorder (Giel et al., 2021). In non‐clinical samples, being female was identified as a risk factor for pandemic‐related binge eating (Flaudias et al., 2020) and feeling more out of control when eating (Robertson et al., 2021), a key component of binge eating. During the pandemic, those suffering from symptoms of other psychiatric disorders and lockdown‐related stress have also been reported to be at increased risk of binge eating (Flaudias et al., 2020). In terms of other eating disorder symptoms, being at a low weight, typically considered to be a body mass index below 18.5 kg/m^2^, is a core symptom of anorexia nervosa (American Psychiatric Association, 2013). A systematic review of studies of both general population participants and patients, for instance, those attending an obesity clinic, found that 11%–32% of participants experienced weight loss during the pandemic; stress and previous low weight were suggested contributing factors (Khan et al., 2021). In line with this, self‐reported reduction in quantities eaten during lockdown has been linked with stress (Flaudias et al., 2020; Herle et al., 2021), as well as with being female (Flaudias et al., 2020; Herle et al., 2021) and higher scores on measurements of depression (Herle et al., 2021).

Data from the COVID‐19 Social Study, which explored the experiences of ~50,000 UK adults during the COVID‐19 pandemic, indicated that over the first 59 weeks of the pandemic, over one‐quarter of participants reported self‐harm ideation and 8% reported they had self‐harmed at least once (Paul & Fancourt, 2021). However, this may not represent population prevalence as the sample was self‐selected (Pierce et al., 2020). In almost 50% of 228 UK patients presenting to hospital after self‐harm, COVID‐related factors such as loneliness and entrapment were identified as contributing factors (Hawton, Lascelles, et al., 2021). Indeed, experience of each pandemic‐related adverse event, such as financial difficulties, being ill with COVID‐19, or the death or illness of a family member or friend, has been associated with an approximately two‐fold higher likelihood of self‐reported self‐harm and a 1.56 times higher odds of self‐reported self‐harm ideation (Paul & Fancourt, 2021). With regards to self‐reported suicidal ideation, being younger (O'Connor et al., 2020; Sueki & Ueda, 2021), gender diverse (Turner et al., 2021), of a lower socioeconomic group (O'Connor et al., 2020) or lower income (Sáiz et al., 2020), having unstable employment (Sueki & Ueda, 2021), and having pre‐existing mental ill‐health (O'Connor et al., 2020; Sáiz et al., 2020; Sueki & Ueda, 2021; Turner et al., 2021) have been associated with higher risk during the pandemic.

Establishing common and distinct risk factors for new onset of eating disorder and self‐harm symptoms during the pandemic may help to identify subgroups that require closer monitoring of their mental health during future pandemics. In addition, disentangling the complex relationship between these symptoms through shared risk and protective factors will contribute to our understanding of the possible mechanisms underlying their comorbidity. We examined the longer‐term effects of the COVID‐19 pandemic based on UK data collected between April 2020 and July 2021. We investigated whether a range of potential risk factors were longitudinally associated with newly occurring binge eating, low weight, suicidal and/or self‐harm ideation, and self‐harm during the pandemic.

We hypothesized that those groups already at risk of disordered eating — such as women (Keel & Forney, 2013) — or of self‐harm thoughts and behaviors — such as younger individuals (Fliege et al., 2009) — would be at higher risk of newly experiencing these symptoms during the pandemic. Furthermore, we hypothesized that individuals who experienced COVID‐related difficulties, for example, the loss of a loved one due to COVID‐19, would also be more at risk of these mental health difficulties during the pandemic. Finally, given the high overlap of self‐harm thoughts and behaviors and eating disorders (Carano et al., 2012; Claes & Muehlenkamp, 2014; Favaro & Santonastaso, 1997; Milos et al., 2004; Swanson et al., 2011; Wright et al., 2009), we hypothesized that there would be common risk factors across all outcomes.

## 2 METHOD

### 2.1 Sample

Participants were from the Repeated Assessment of Mental health in Pandemics (RAMP) study (*n* = 12,175) and a subsample of the National Institute for Health and Care Research (NIHR) BioResource that joined the COVID‐19 Psychiatry and Neurological Genetics (COPING) study (*n* = 32,896).

#### 2.1.1 RAMP Study

The RAMP Study was set up in April 2020 to better understand the impact of the COVID‐19 pandemic on the mental health and well‐being of UK residents. Recruitment was entirely online, via a social media campaign. Participants completed a baseline assessment and subsequent follow‐up questionnaires fortnightly until July 2020 and then monthly. The RAMP Study questionnaires largely mirror those in the COPING study. Our study includes all RAMP Study data collected between April 2020 and July 2021.

#### 2.1.2 NIHR BioResource

The NIHR BioResource is a databank and recontactable resource of volunteers who have provided medical, clinical, and biological data. Volunteers were recruited through a variety of approaches, including National Health Service (NHS) blood transfusion services and various disease/disorder focussed research efforts.

Throughout the pandemic, NIHR BioResource participants were given the opportunity to join the COPING study, which launched in April 2020. The COPING study contained questionnaires from the sign‐up surveys of the Genetic Links to Anxiety and Depression (GLAD) Study (Davies et al., 2019) and the Eating Disorders Genetics Initiative (EDGI UK), as well as additional questionnaires to assess COVID‐related variables, that is, experiences related to the COVID‐19 pandemic. Participants first completed a baseline survey and then follow‐up surveys, initially every two weeks but then monthly from August 2020. The first COPING study baseline survey was distributed to existing NIHR BioResource participants on the 30th April 2020, at which point stay‐at‐home orders had been in place for approximately one month (i.e., since 26th March 2020). All invites for existing participants were sent by 11th May 2020. New GLAD Study and EDGI UK survey participants were sent invitations throughout the pandemic. The last round of invites was sent on 19th January 2021. Details are described elsewhere (Davies et al., 2022). Our study includes all COPING study data collected between April 2020 and July 2021.

COPING study participants come from multiple sub‐cohorts of the NIHR BioResource, including: the GLAD Study (*n* = 14,948); EDGI UK (*n* = 1,010); the Inflammatory Bowel Disease BioResource (*n* = 3,203); NHS blood and transplant studies, including INTERVAL (*n* = 4,656), COMPARE (*n* = 1,928), and STRategies to Improve Donor Experiences (STRIDES; *n* = 2,808); and the Research Tissue Bank—Generic (*n* = 4,343). Saliva samples for DNA have not been provided by all participants of the GLAD Study or EDGI UK and therefore some participants are not full members of either study. Therefore, we refer to these participants as GLAD Study survey participants and EDGI UK survey participants. Further detail about these cohorts is in Table 1, with numbers of participants after exclusion.

**TABLE 1:** The Repeated Assessment of Mental health in Pandemics (RAMP) Study and the COPING study participants divided by the sub‐cohorts of the National Institute for Health and Care Research (NIHR) BioResource comprising the analysis samples (*n* = 36,715)

	*N*	Recruitment methods	Eligibility criteria	Recruitment area
Repeated Assessment of Mental health in Pandemics (RAMP) Study	10,026	Social media	16+ years, live in the UK	England, Wales, Scotland, Northern Ireland
Genetic Links to Anxiety and Depression (GLAD) Study	10,779	Social media, NHS recruitment sites	16+ years, live in the UK, lifetime experience of anxiety and/or depression	England, Wales, Scotland, Northern Ireland
Eating Disorders Genetics Initiative (EDGI UK)	502	Social media	16+, live in England, have lifetime experience of any eating disorder	England
Inflammatory Bowel Disease (IBD) cohort	2,714	IBD clinics in participating hospitals across the UK	16+, have a diagnosis of Crohn's disease, ulcerative colitis, indeterminate colitis, IBD type unspecified, or suspected IBD	England, Wales, Scotland, Northern Ireland
NHS Blood and Transplant studies (COMPARE, STRIDES, INTERVAL)	8,766	Blood donation centers	16+, live in England	England
Research Tissue Bank—Generic (RTB‐GEN)	3,928	Biomedical Research Centers, Clinical Research Facilities, hospital clinics, community recruitment, online	16+, live in England	England

### 2.2 Ethical approval

The London—Fulham Research Ethics Committee approved the GLAD Study on 21st August 2018 (REC reference: 18/LO/1218) and EDGI UK on 29th July 2019 (REC reference: 19/LO/1254). The NIHR BioResource has been approved as a Research Tissue Bank by the East of England—Cambridge Central Committee (REC reference: 17/EE/0025). The COVID‐19 Psychiatry and Neurological Genetics study was approved by the South West—Central Bristol Research Ethics Committee on 27th April 2020 (REC reference: 20/SW/0078). The RAMP Study was approved by the Psychiatry, Nursing, and Midwifery Research Ethics Committee at King's College London on 27th March 2020 (HR‐19/20–18157).

### 2.3 Measures

A detailed summary of how we defined exposure and outcome variables is in the Supplementary materials. We defined each “phase” as the time period starting from the date one survey was distributed and ending the day before the next survey was distributed.

#### 2.3.1 Exposures

*Socio‐demographic variables*: Socio‐demographic information including age, race/ethnicity, sex, gender, and employment status was collected from the COPING study and RAMP Study baseline surveys, and the GLAD Study and EDGI UK sign‐up surveys. We collapsed race and gender categories into binary variables of “racially minoritized” and “minoritized gender,” respectively. ​Sample sizes in the more refined racial and gender groups were too small to keep them as independent categories.

*Psychiatric disorders*: We assessed lifetime mental health diagnoses via the Mental Health Diagnosis questionnaire (MHD), adapted from the UK Biobank Questionnaire (Davis et al., 2020). This includes questions such as: “*Have you been diagnosed with one or more of the following mental health problems by a professional, even if you don't have it currently?*”, followed by a list of psychiatric disorders. The MHD was included in the RAMP Study baseline survey and the GLAD Study and EDGI UK sign‐up surveys. The MHD was also included in the baseline COPING study questionnaire for all sub‐cohorts other than EDGI UK because the launch dates of EDGI UK and the COPING study were less than  months apart. We additionally identified participants with algorithm‐derived lifetime eating disorder diagnoses via responses to the ED100K (Thornton et al., 2018) (an eating disorder questionnaire which asks questions about a range of DSM‐5 [American Psychiatric Association, 2013] eating disorder symptoms).

*COVID‐related variables*. We measured pandemic worry scores (via a non‐validated 21‐item scale developed by the RAMP Study team), being a vulnerable group member, and pandemic‐related loneliness in the COPING study and the RAMP Study baseline surveys.

The remaining exposure variables were measured beyond the baseline assessment, that is, at frequent intervals during the monitoring period. This included: COVID‐19 illness or a positive test, loss of a loved one or relative due to COVID‐19, change in main economic activity (work or education), and change in living situation. We only included instances in which the first report of the exposure occurred in the same phase as or in a phase before the first report of the outcome.

#### 2.3.2 Outcomes

Participants with no pre‐pandemic experience of each outcome form the basis of our analysis. Participants self‐reported pre‐pandemic experience of self‐harm‐related outcomes in the COPING study and RAMP Study baseline surveys (e.g., *“Many people have thoughts that life is not worth living. Had you felt that way before the pandemic?*”). Contrastingly, participants were not explicitly asked about their pre‐pandemic experience of binge eating or low weight. GLAD Study and EDGI UK survey participants who answered the relevant sign‐up questionnaire up to three months before the pandemic and who did not endorse lifetime low weight and/or binge eating or a related lifetime diagnosis were classified as not having pre‐pandemic experience. For participants who endorsed lifetime low weight and/or binge eating in the COPING study baseline survey (which launched during the pandemic), we cross‐checked their age at symptom start with their age at the start of the pandemic. Participants for whom their age at symptom start was older than their age at the start of the pandemic were classified as not having pre‐pandemic experience of the symptom. At baseline, RAMP Study participants self‐reported diagnoses of anorexia nervosa, psychological overeating or binge‐eating disorder, and bulimia nervosa, as well as binge eating in the 28 days before the pandemic. RAMP Study participants were not asked about their experiences of low weight at baseline. Therefore, RAMP Study participants without a diagnosis of anorexia nervosa were classified as not having pre‐pandemic experience of low weight. We do not equate low weight with anorexia nervosa, however, this was the only available measure for low weight in the RAMP Study. RAMP Study participants without a binge‐type eating disorder diagnosis at baseline and who indicated they did not binge eat in the 28 days before the pandemic were classified as not having pre‐pandemic experience of binge eating (see Supplementary materials for full details).

*New‐onset binge eating*: Binge eating was assessed via a screener question to the ED100K (Thornton et al., 2018) included at baseline and every other follow‐up survey in the RAMP Study and the COPING study survey: “*Over the past month, have you had regular episodes of overeating or eating binges when you ate what most people would regard as an unusually large amount of food in a short period of time?*” Participants with no pre‐pandemic experience of binge eating and who endorsed binge eating at any point during the monitoring period were classified as having new‐onset binge eating.

*New‐onset low weight*: Low weight was assessed via the ED100K (Thornton et al., 2018) screener question included at baseline and every other follow‐up in the RAMP Study and the COPING study: “*Over the past month, have you weighed much less than other people thought you ought to weigh?*”. Again, participants with no pre‐pandemic experience of low weight and who, at any point during the monitoring period, endorsed low weight were classified as having new‐onset low weight.

*New‐onset suicidal and/or self‐harm ideation*: The thoughts and feelings questionnaire (TAF) was adapted from the UK Biobank (Davis et al., 2020) and was included at baseline and every follow‐up in the RAMP Study and the COPING study. The TAF includes questions investigating pre‐pandemic, lifetime, and recent (past 2 weeks) passive suicidal ideation (e.g., “*Many people have thoughts that life is not worth living. Have you felt that way in the past two weeks?*”) and self‐harm ideation (e.g., “*Have you contemplated harming yourself in the past two weeks?*”). We combined “suicidal ideation” and “self‐harm ideation” because pre‐processing checks revealed a correlation of 0.86 (Supplementary Figure S1).

Participants with no pre‐pandemic experience of either symptom and who then endorsed recent or lifetime suicidal and/or self‐harm ideation during the monitoring period were categorized as having new‐onset suicidal and/or self‐harm ideation.

*New‐onset self‐harm*: The question “*In the last two weeks, have you deliberately harmed yourself, whether or not you meant to end your life?*” was included in the TAF at baseline and every follow‐up. Participants with no pre‐pandemic experience of self‐harm and who endorsed self‐harm during the monitoring period were classified as having new‐onset self‐harm.

For all exposures, participants who answered *“No”* at least once and never answered *“Yes”* to experiencing the relevant outcome were categorized as having not experienced the outcome during the monitoring period.

### 2.4 Exclusion criteria

We excluded a total of 8,356 participants with missing data on all exposure variables and/or all outcome variables, leaving a total of 36,715 participants for inclusion in data analysis. Significantly more excluded participants were in the age bracket of 36–45 years or younger, female, of a minoritized gender, racially minoritized, of lower education status, and had significantly lower body mass indexes (BMIs) at registration (Table S1).

### 2.5 Data analysis

We conducted all analyses in R version 4.1.2 (R Core Team, R., & Others, 2013). All code for this study is publicly available: https://github.com/RAMP-COPING/EDBehaviour_SelfHarm.

*Pre‐processing*: We assessed multicollinearity by calculating the correlation between all exposure variables included in each regression model (Figures S2–S5). Correlations above 0.7 violated our assumption of no multicollinearity.

*Descriptives*: First, we described the age, sex, gender, race/ethnicity, BMI (at registration), and education level of the overall sample (*n* = 36,715) and each subsample with complete data on at least one relevant variable, defined by outcome: binge eating (*n* = 24,211), low weight (*n* = 24,364), suicidal and/or self‐harm ideation (*n* = 18,040), and self‐harm (*n* = 29,948). Second, we described the percentage of participants in each subsample that self‐reported new onset of each symptom during the monitoring period, both in the main analysis and all sensitivity analyses (Table S2).

*Regression analyses*: We fitted multiple logistic regression models to estimate associations between our exposure variables and new onset of (1) binge eating, (2) low weight, (3) suicidal and/or self‐harm ideation, and (4) self‐harm. We used the *glm* function from the *stats* package to regress each outcome onto each exposure variable, including appropriate covariates. We plotted results using *ggplot*. We controlled for multiple testing by using a *p*‐value threshold adjusted for the total number of variables included in each model $(\alpha =\frac{0.05}{13}=0.0038$).

*Additional analyses*: We conducted an additional analysis to explore education as a risk factor, in which we excluded participants aged 16–25 years because their education may not have been finished at that timepoint. We also examined the interaction between sex and age by conducting age‐stratified regressions and reporting the association between sex and each outcome.

*Sensitivity analyses*: First, we restricted our analyses to participants who were not originally ascertained for psychiatric disorders (i.e., excluded GLAD Study and EDGI UK survey participants). In a second sensitivity analysis, we excluded all participants from the IBD sub‐cohort and those who self‐reported a diagnosis of IBD. We conducted a third sensitivity analysis in which we specified that participants must have answered “*No*” at least three times to be categorized as having not experienced the relevant outcome, to assess the influence of missing data. In a final sensitivity analysis, we dropped all instances in which the exposure was first reported in the same phase as the outcome, with the aim to further limit the possibility of the outcome impacting the exposures.

## 3 RESULTS

### 3.1 Descriptives

Both the entire sample and every subsample defined by outcome consisted of majority white (~94%), female (64%–70%), and highly educated (e.g., over 50% of participants had at least a Bachelor's degree) participants (Table 2). The most common age category was 56–65 years (25%–28%). A breakdown of gender and race/ethnicity categories can be found in Table 2. Within each subsample, new onset was reported by: 21% for binge eating, 10.8% for low weight, 23.5% for suicidal and/or self‐harm ideation, and 3.5% for self‐harm. In our sensitivity analyses, these percentages changed only marginally (Table S2).

**TABLE 2:** Characteristic of the sample after exclusion (*n* = 36,715) and of each subsample defined by outcome: Binge eating (*n* = 24,211), low weight (*n* = 24,364), suicidal and/or self‐harm ideation (*n* = 18,040), and self‐harm (*n* = 29,948)

	Sample after exclusion	Binge eating	Low weight	Suicidal and/or self‐harm ideation	Self‐harm
Total (*n*)	36,715	24,211	24,364	18,040	29,948
Age (years)					
16–25	3,184 (8.7%)	1,752 (7.2%)	1,897 (7.8%)	964 (5.3%)	1,806 (6.0%)
26–35	4,867 (13.3%)	2,538 (10.5%)	2,959 (12.1%)	1,829 (10.1%)	3,270 (10.9%)
36–45	5,005 (13.6%)	2,836 (11.7%)	3,018 (12.4%)	2,053 (11.4%)	3,769 (12.6%)
46–55	7,559 (20.6%)	4,847 (20.0%)	4,980 (20.4%)	3,603 (20.0%)	6,308 (21.1%)
56–65	9,057 (24.7%)	6,624 (27.4%)	6,330 (26.0%)	5,015 (27.8%)	8,143 (27.2%)
66–70	3,640 (9.9%)	2,869 (11.8%)	2,661 (10.9%)	2,326 (12.9%)	3,409 (11.4%)
71+	3,401 (9.3%)	2,745 (11.3%)	2,519 (10.3%)	2,250 (12.5%)	3,241 (10.8%)
Missing	<5 (<0.1%)	0	0	0	<5 (<0.1%)
Assigned sex at birth					
Female	25,728 (70.1%)	16,304 (67.3%)	16,693 (68.5%)	11,626 (64.4%)	20,107 (67.1%)
Male	10,874 (29.6%)	7,833 (32.4%)	7,578 (31.1%)	6,389 (35.4%)	9,780 (32.7%)
Missing	113 (0.3%)	74 (0.3%)	93 (0.4%)	25 (0.1%)	61 (0.2%)
Gender					
Woman	25,418 (69.2%)	16,154 (66.7%)	16,512 (67.8%)	11,587 (64.2%)	19,988 (66.8%)
Man	10,863 (29.6%)	7,819 (32.3%)	7,572 (31.1%)	6,376 (35.3%)	9,747 (32.5%)
Prefer to self‐define	61 (0.2%)	31 (0.1%)	44 (0.2%)	12 (0.1%)	34 (0.1%)
Non‐binary	303 (0.8%)	166 (0.7%)	191 (0.8%)	50 (0.3%)	141 (0.5%)
Missing	70 (0.2%)	41 (0.2%)	45 (0.2%)	15 (0.1%)	38 (0.1%)
Transgender					
Yes	253 (0.7%)	152 (0.6%)	184 (0.8%)	43 (0.2%)	109 (0.4%)
No	21,033 (57.3%)	12,641 (52.2%)	13,962 (57.3%)	7,044 (39.1%)	15,434 (51.5%)
Missing^a^	15,429 (42.0%)	11,418 (47.2%)	10,218 (41.9%)	10,953 (60.7%)	14,405 (48.1%)
Education level					
No formal qualifications	1034 (2.8%)	679 (2.8%)	639 (2.6%)	595 (3.3%)	919 (3.1%)
GCSE/CSE or equivalent	4,134 (11.3%)	2,713 (11.2%)	2,706 (11.1%)	2,052 (11.4%)	3,369 (11.2%)
NVQ, HND, HNC or equivalent	2,064 (5.6%)	1,312 (5.4%)	1,200 (4.9%)	1,193 (6.6%)	1,782 (6.0%)
A‐Levels or equivalent	5,791 (15.8%)	3,568 (14.7%)	3,733 (15.3%)	2,485 (13.8%)	4,410 (14.7%)
Other professional qualification	2,838 (7.7%)	1,940 (8.0%)	1,817 (7.5%)	1,665 (9.2%)	2,469 (8.2%)
Bachelor's degree or equivalent	10,898 (29.7%)	7,240 (29.9%)	7,458 (30.6%)	5,222 (28.9%)	8,816 (29.4%)
Master's degree or equivalent	4,213 (11.5%)	2,858 (11.8%)	2,902 (11.9%)	1,986 (11.0%)	3,407 (11.4%)
Postgraduate degree or equivalent	4,268 (11.6%)	2,846 (11.8%)	2,914 (12.0%)	2,090 (11.6%)	3,548 (11.8%)
PhD	1,454 (4.0%)	1,045 (4.3%)	985 (4.0%)	741 (4.1%)	1,210 (4.0%)
Missing	21 (0.1%)	10 (<0.1%)	10 (<0.1%)	11 (0.1%)	18 (0.1%)
Race/ethnicity					
Arab	18 (<0.1%)	9 (<0.1%)	12 (<0.1%)	5 (<0.1%)	13 (<0.1%)
Asian	442 (1.2%)	257 (1.1%)	249 (1.0%)	217 (1.2%)	358 (1.2%)
Black	188 (0.5%)	116 (0.5%)	126 (0.5%)	92 (0.5%)	157 (0.5%)
Mixed race	488 (1.3%)	268 (1.1%)	298 (1.2%)	174 (1.0%)	334 (1.1%)
Other	212 (0.6%)	115 (0.5%)	122 (0.5%)	78 (0.4%)	165 (0.6%)
White	34,641 (94.4%)	22,932 (94.7%)	23,055 (94.6%)	17,002 (94.2%)	28,264 (94.4%)
Missing	726 (2.0%)	514 (2.1%)	502 (2.1%)	472 (2.6%)	657 (2.2%)
BMI [kg/m^2^] (median, IQR)^b^					
At registration	29.0 (8.3)	28.2 (7.2)	29.9 (8.3)	28.7 (7.4)	28.9 (7.8)

*Note*: Participants are from the National Institute for Health and care Research (NIHR) BioResource sub‐cohorts who joined the COVID‐19 Psychiatry and Neurological Genetics (COPING) study, or the Repeated Assessment of Mental health in Pandemics (RAMP) Study. Participants reported new onset of each symptom in the COPING study or the RAMP Study.

Abbreviations: BMI, body mass index; GCSE/CSE, General Certificate of Secondary Education/Certificate of Secondary Education; HNC, Higher National Certificates; HND, Higher National Diplomas, IQR, interquartile range; NVQ, National Vocational Qualification.

^a^ The high missingness of the transgender variable is because this question was not included in all surveys.

^b^ BMI data were not normally distributed (Figure S6) so we reported median and IQR.

**TABLE 3:** Logistic regression results for each outcome (binge eating *n* = 24,211; low weight *n* = 24,364; suicidal and/or self‐harm ideation *n* = 18,040; self‐harm *n* = 29,948), including details on the included covariates for each exposure

	Binge eating		Low weight		Suicidal and/or self‐harm ideation		Self‐harm		Covariates
Exposure	OR (95% CIs)	*p*‐value	OR (95% CIs)	*p*‐value	OR (95% CIs)	*p*‐value	OR (95% CIs)	*p‐v*alue	
Age									No covariates^a^
16–25 years	1.0 (0.88, 1.13)	0.96	1.34 (1.15, 1.56)	2.04 × 10^−4^	1.90 (1.64, 2.21)	6.03 × 10^−17^	2.64 (2.13, 3.26)	3.45 × 10^−9^	
26–35 years	0.95 (0.85, 1.06)	0.36	0.69 (0.59, 0.81)	2.77 × 10^−6^	1.33 (1.17, 1.5)	1.08 × 10^−5^	1.93 (1.59, 2.34)	3.59 × 10^−11^	
36–45 years	1.01 (0.9, 1.12)	0.88	0.87 (0.75, 1.01)	0.07	1.07 (0.94, 1.21)	0.32	1.21 (0.98, 1.5)	0.07	
56–65 years	0.82 (0.75, 0.89)	8.56 × 10^−6^	0.99 (0.88, 1.11)	0.83	0.91 (0.82, 1)	0.06	0.66 (0.54, 0.8)	3.92 × 10^−5^	
66–70 years	0.62 (0.55, 0.7)	2.62 × 10^−15^	0.82 (0.7, 0.96)	0.01	0.65 (0.57, 0.74)	7.76 × 10^−11^	0.5 (0.37, 0.66)	2.63 × 10^−6^	
71+ years	0.53 (0.46, 0.6)	1.37 × 10^−23^	0.95 (0.82, 1.11)	0.53	0.67 (0.58, 0.76)	2.30 × 10^−9^	0.43 (0.31, 0.58)	1.12 × 10^−7^	
Female	1.55 (1.44, 1.66)	3.34 × 10^−34^	0.92 (0.84, 1)	0.06	1.95 (1.81, 2.11)	1.96 × 10^−63^	1.5 (1.3, 1.74)	2.58 × 10^−8^	No covariates^a^
Racially minoritized	1.48 (1.26, 1.74)	1.45 × 10^−6^	1.43 (1.17, 1.74)	4.56 × 10^−4^	1.66 (1.39, 1.98)	2.01 × 10^−8^	1.4 (1.03, 1.86)	0.02	No covariates
Minoritized gender	1.27 (0.97, 1.65)	0.07	1.22 (0.88, 1.65)	0.23	3.66 (2.43, 5.55)	6.34 × 10^−10^	2.08 (1.3, 3.15)	1.14 × 10^−3^	Age
Psychiatric disorder	1.87 (1.74, 2.01)	2.66 × 10^−62^	1.62 (1.47, 1.78)	7.38 × 10^−22^	4.61 (4.23, 5.03)	4.95 × 10^−264^	2.67 (2.25, 3.17)	5.37 × 10^−29^	Age, sex, minoritized gender, racially minoritized, vulnerable group member, COVID‐19 illness and/or positive test, lost someone due to COVID‐19
Employment									Age, sex, racially minoritized, minoritized gender, psychiatric disorder, vulnerable group member
Key worker	1.29 (1.18, 1.4)	5.91 × 10^−9^	1.23 (1.09, 1.38)	6.21 × 10^−4^	0.97 (0.88, 1.07)	0.57	1.03 (0.88, 1.22)	0.69	
Not in paid employment	1.46 (1.3, 1.64)	2.11 × 10^−10^	1.56 (1.35, 1.81)	3.10 × 10^−9^	1.40 (1.2, 1.63)	1.38 × 10^−5^	1.54 (1.24, 1.91)	6.94 × 10^−5^	
Retired	0.97 (0.86, 1.09)	0.61	1.02 (0.87, 1.19)	0.8	1.0 (0.87, 1.14)	0.95	0.99 (0.75, 1.31)	0.95	
Student	1.2 (0.98, 1.47)	0.08	1.75 (1.37, 2.23)	6.07 × 10^−6^	1.03 (0.79, 1.34)	0.82	1.34 (0.96, 1.85)	0.08	
Vulnerable group member	1.35 (1.25, 1.46)	4.35 × 10^−14^	1.81 (1.64, 1.99)	1.35 × 10^−32^	1.17 (1.08, 1.28)	3.44 × 10^−4^	1.26 (1.08, 1.46)	3.16 × 10^−3^	Sex, age
Pandemic worry									Age, sex, racially minoritized, psychiatric disorder, employment, vulnerable group member, lost someone due to COVID‐19
21–40	1.46 (1.34, 1.6)	7.78 × 10^−17^	1.34 (1.19, 1.51)	1.05 × 10^−6^	1.58 (1.43, 1.75)	3.50 × 10^−19^	1.24 (1.02, 1.51)	0.03	
41–60	2.29 (2.05, 2.56)	4.23 × 10^−49^	2.06 (1.78, 2.37)	2.74 × 10^−23^	2.55 (2.23, 2.92)	1.20 × 10^−41^	1.77 (1.42, 2.22)	4.82 × 10^−7^	
61–80	2.97 (2.16, 4.07)	1.32 × 10^−11^	3.29 (2.36, 4.54)	8.90 × 10^−13^	3.84 (2.52, 5.9)	4.99 × 10^−10^	2.76 (1.71, 4.29)	1.51 × 10^−5^	
Increased loneliness during pandemic	1.14 (1.05, 1.23)	1.01 × 10^−3^	1.03 (0.93, 1.14)	0.54	1.29 (1.18, 1.42)	6.63 × 10^−8^	1.18 (1.01, 1.38)	0.03	Age, sex, psychiatric disorder, employment, vulnerable group member, pandemic worry score, COVID‐19 illness and/or positive test, lost someone due to COVID‐19, change in main economic activity, change in living situation
Lost someone due to COVID‐19	0.93 (0.82, 1.05)	0.25	1.01 (0.87, 1.18)	0.87	0.70 (0.6, 0.82)	1.53 × 10^−5^	1.21 (0.95, 1.52)	0.12	Age, sex, racially minoritized, vulnerable group member
Change in main economic activity	1.01 (0.94, 1.09)	0.76	1.05 (0.95, 1.16)	0.37	1.17 (1.07, 1.28)	9.08 × 10^−4^	1.22 (1.05, 1.41)	8.7 × 10^−3^	Age, sex, racially minoritized, psychiatric disorder, employment, vulnerable group member
Change in living situation	0.79 (0.73, 0.86)	1.42 × 10^−7^	0.89 (0.79, 0.99)	0.03	0.71 (0.63, 0.79)	3.51 × 10^−9^	1.07 (0.9, 1.26)	0.44	Age, sex, racially minoritized, psychiatric disorder, employment, vulnerable group member, pandemic worry, change in main economic activity
COVID‐19 illness and/or positive test	0.38 (0.3, 0.47)	2.21 × 10^−18^	0.54 (0.42, 0.69)	1.61 × 10^−6^	0.24 (0.17, 0.33)	1.83 × 10^−17^	0.61 (0.4, 0.89)	0.02	Age, sex, racially minoritized, employment, vulnerable group member, pandemic worry score, change in main economic activity, change in living situation

*Note*: Minoritized gender, “Transgender,” “Non‐binary,” and “Prefer to self‐define.” Racially minoritized, “Arab,” “Asian,” “Black,” “Mixed race,” and “Other.” The exact definition of all other variables are in the Supplementary materials. Reference category for age is 46–55 years, for female is being male, for racially minoritized is being white, for minoritized gender is not being minoritized gender, for psychiatric disorder is not having a psychiatric disorder, for employment is being in paid employment, for vulnerable group member is not being a vulnerable group member, for pandemic worry score is a score of 0–20, for losing someone due to COVID‐19 is not losing someone due to COVID‐19, for change in main economic activity is not experiencing a change in main economic activity, and for change in living situation is not experiencing a change in living situation.

^a^ We conducted an additional analysis in which we investigated sex by age effects.

### 3.2 Regression analyses results

All results are shown in Table 3. Below we have categorized risk and protective factors as “shared” (i.e., across eating disorder and self‐harm symptoms) or “specific” (i.e., relating to either eating disorder or self‐harm symptoms). We report only on significant associations. For each subsample, we have provided information on the *n* of each exposure split by outcome in Tables S3–S6.

#### 3.2.1 Shared risk factors

**FIGURE 1:**
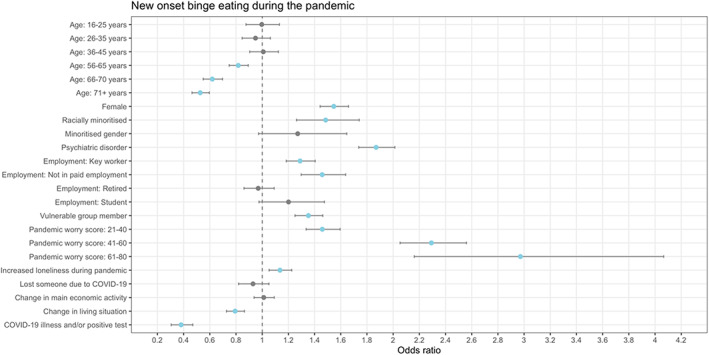
Association between demographic and COVID‐related variables and new onset of binge eating during the pandemic derived from multiple logistic regression models (*n* = 24,211). Points in blue are significant (*p* < .0038). Minoritized gender = “Transgender,” “Non‐binary,” and “Prefer to self‐define”; Racially minoritized = “Arab,” “Asian,” “Black,” “Mixed race,” and “Other.” The exact definition of all other variables are in the Supplementary materials. Information on covariates is in Table 3. Reference category for age is 46–55 years, for female is being male, for racially minoritized is being white, for minoritized gender is not being a minoritized gender, for psychiatric disorder is not having a psychiatric disorder, for employment is being in paid employment, for vulnerable group member is not being a vulnerable group member, for pandemic worry score is a score of 0–20, for losing someone due to COVID‐19 is not losing someone due to COVID‐19, for change in main economic activity is not experiencing a change in main economic activity, and for change in living situation is not experiencing a change in living situation.

**FIGURE 2:**
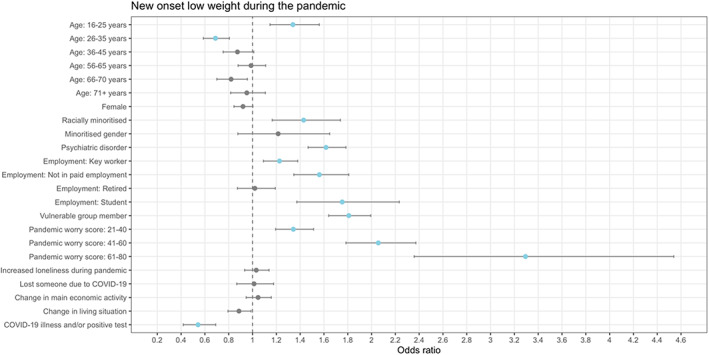
Association between demographic and COVID‐related variables and new onset of low weight during the pandemic derived from multiple logistic regression models (*n* = 24,364). Points in blue are significant (*p* < .0038). Minoritized gender = “Transgender,” “Non‐binary,” and “Prefer to self‐define”; Racially minoritized = “Arab,” “Asian,” “Black,” “Mixed race,” and “Other.” The exact definition of all other variables are in the Supplementary materials. Information on covariates is in Table 3. Reference category for age is 46–55 years, for female is being male, for racially minoritized is being white, for minoritized gender is not being a minoritized gender, for psychiatric disorder is not having a psychiatric disorder, for employment is being in paid employment, for vulnerable group member is not being a vulnerable group member, for pandemic worry score is a score of 0–20, for losing someone due to COVID‐19 is not losing someone due to COVID‐19, for change in main economic activity is not experiencing a change in main economic activity, and for change in living situation is not experiencing a change in living situation.

**FIGURE 3:**
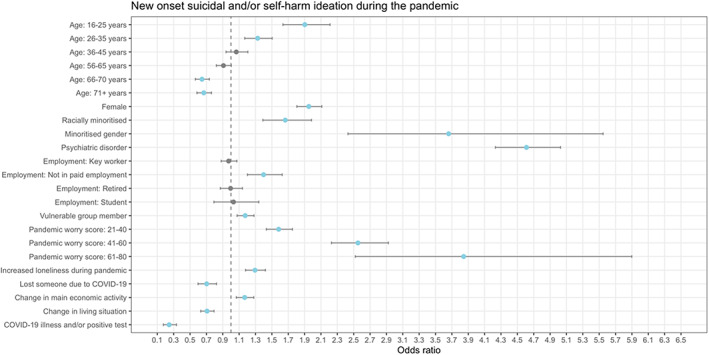
Association between demographic and COVID‐related variables and new onset of suicidal and/or self‐harm ideation during the pandemic derived from multiple logistic regression models (*n* = 18,040). Points in blue are significant (*p* < .0038). Minoritized gender = “Transgender,” “Non‐binary,” and “Prefer to self‐define”; Racially minoritized = “Arab,” “Asian,” “Black,” “Mixed race,” and “Other.” The exact definition of all other variables are in the Supplementary materials. Information on covariates is in Table 3. Reference category for age is 46–55 years, for female is being male, for racially minoritized is being white, for minoritized gender is not being a minoritized gender, for psychiatric disorder is not having a psychiatric disorder, for employment is being in paid employment, for vulnerable group member is not being a vulnerable group member, for pandemic worry score is a score of 0–20, for losing someone due to COVID‐19 is not losing someone due to COVID‐19, for change in main economic activity is not experiencing a change in main economic activity, and for change in living situation is not experiencing a change in living situation

**FIGURE 4:**
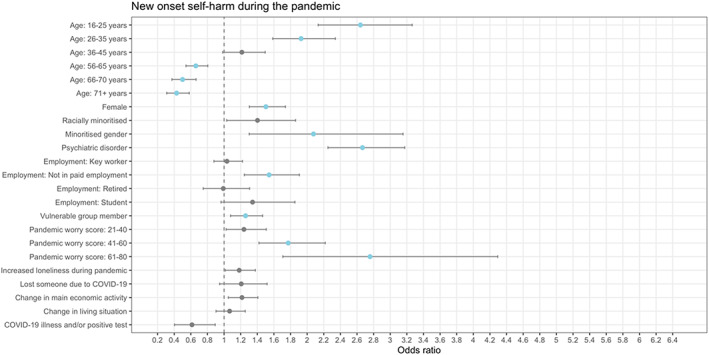
Association between demographic and COVID‐related variables and new onset of self‐harm during the pandemic derived from multiple logistic regression models (*n* = 29,948). Points in blue are significant (*p* < .0038). Minoritized gender = “Transgender,” “Non‐binary,” and “Prefer to self‐define”; Racially minoritized = “Arab,” “Asian,” “Black,” “Mixed race,” and “Other.” The exact definition of all other variables are in the Supplementary materials. Information on covariates is in Table 3. Reference category for age is 46–55 years, for female is being male, for racially minoritized is being white, for minoritized gender is not being a minoritized gender, for psychiatric disorder is not having a psychiatric disorder, for employment is being in paid employment, for vulnerable group member is not being a vulnerable group member, for pandemic worry score is a score of 0–20, for losing someone due to COVID‐19 is not losing someone due to COVID‐19, for change in main economic activity is not experiencing a change in main economic activity, and for change in living situation is not experiencing a change in living situation

A lifetime psychiatric disorder was associated with higher odds of new‐onset binge eating (Figure 1), low weight (Figure 2), suicidal and/or self‐harm ideation (Figure 3), and self‐harm (Figure 4) during the pandemic. Another shared risk factor was pandemic worry; higher pandemic worry scores were associated with higher odds of all outcomes. Compared to being in paid employment, not being in paid employment was linked with higher odds of all outcomes. Being a member of a vulnerable group, such as an organ transplant recipient, was linked to higher odds of all outcomes. Being female was a risk factor for binge eating, suicidal and/or self‐harm ideation, and self‐harm. Self‐reporting greater loneliness during the pandemic than before the pandemic was linked to higher odds of binge eating and suicidal and/or self‐harm ideation. Compared to being in mid‐life (46–55 years), being aged 16–25 years was associated with higher odds of low weight, suicidal and/or self‐harm ideation, and self‐harm. Being racially minoritized was a risk factor for binge eating, low weight, and suicidal and/or self‐harm ideation.

#### 3.2.2 Shared protective factors

A shared protective factor was a change in living situation, which was associated with lower odds of binge eating and suicidal and/or self‐harm ideation. We also found that infection with COVID‐19 was associated with lower odds of binge eating, low weight, and suicidal and/or self‐harm ideation. Compared to participants in mid‐life, the odds of binge eating, suicidal and/or self‐harm ideation, and self‐harm were lower in older participants, specifically those aged 66–70 and 71+ years across all these  outcomes and additionally 56–65 years for binge eating and self‐harm.

#### 3.2.3 Specific risk factors

Compared to being in mid‐life, being aged 26–35 years was associated with higher odds of suicidal and/or self‐harm ideation and self‐harm. Compared to being in other paid employment, being a key worker, such as working in health and social care, was linked to higher odds of binge eating and low weight, and being a student was associated with higher odds of low weight. Self‐reporting a change in main economic activity, for example, becoming unemployed or experiencing a change in duties or responsibilities, was associated with higher odds of suicidal and/or self‐harm ideation. Being a minoritized gender was a risk factor for suicidal and/or self‐harm ideation and self‐harm.

#### 3.2.4 Specific protective factors

Loss of a loved one or relative due to COVID‐19 was associated with a reduction in the odds of suicidal and/or self‐harm ideation. Compared to being in mid‐life, being aged 26–35 years was associated with lower odds of low weight.

### 3.3 Additional analyses results

#### 3.3.1 Highest education

As shown in Table SA1, compared to having no formal qualifications, being of a higher education status (NVQ, HND, HNC or equivalent; A‐levels or equivalent; or university/professional qualification) was associated with lower odds of binge eating (OR = 0.62, 95% CI 0.49, 0.79, *p* < .001; OR = 0.70, 95% CI 0.57, 0.87, *p* < .001; OR = 0.56, 95% CI 0.46, 0.68, *p* < .001). Similarly, being of a higher education status (GCSE/CSE or equivalent; NVQ, HND, HNC or equivalent; A‐levels or equivalent; or university/professional qualification) was associated with lower odds of low weight (OR = 0.66, 95% CI 0.51, 0.85, *p* = .001; OR = 0.62, 95% CI 0.47, 0.82, *p* < .001; OR = 0.64, 95% CI 0.50, 0.82, *p* < .001; OR = 0.47, 95% CI 0.38, 0.59, *p* < .001). We found no other significant associations.

#### 3.3.2 Sex by age interaction

The association between being female and suicidal and/or self‐harm ideation was greatest in younger participants (aged 16–25 years; OR = 2.75, 95% CI 1.85, 4.20, *p* < .001) and in older participants (aged 66–70 years; OR = 2.55, 95% CI 2.02, 3.24, *p* < .001), indicating an interaction between age and sex. Furthermore, the association between being female and binge eating was greatest in participants older than mid‐life. For example, being aged 56–65 years old was associated with 1.68 higher odds of binge eating (95% CI 1.48, 1.92, *p* < .001). We found no other significant associations (see Table SA2 for full details).

### 3.4 Sensitivity analyses results

Our sensitivity analyses indicated that our results from the main analysis were robust to ascertainment bias in terms of psychiatric disorders (Figures S7–S10) and inflammatory bowel disease (Figures S11–S14), missing data (Figures S15–S18), and our attempt to further limit the possibility of reverse causality (Figures S19–S22) (see Table SBs for further details).

## 4 DISCUSSION

One of the strongest shared risk factors for new onset of all outcomes in our study — binge eating, low weight, suicidal and/or self‐harm ideation, and self‐harm — was a lifetime psychiatric disorder, consistent with previous research (Flaudias et al., 2020; Herle et al., 2021; O'Connor et al., 2020; Sáiz et al., 2020; Sueki & Ueda, 2021; Turner et al., 2021). Experiencing mental health challenges has been reported to result in reduced resilience in the face of adversity (Verdolini et al., 2021), leaving a person vulnerable to experiencing further psychiatric symptoms (Killgore et al., 2020). In addition, participants in our study with a pre‐existing psychiatric disorder may represent a subgroup of individuals more vulnerable to adverse mental health because of, for example, their genetics or prior exposure to traumatic events. Therefore, these individuals may have been more likely to experience new psychiatric symptoms during extreme changes in their environment, such as during a pandemic. Another shared risk factor across all outcomes was pandemic‐associated worry. This is consistent with our hypothesis that individuals with pandemic‐related difficulties would be more at risk of psychiatric symptoms. Indeed, worries specific to the COVID‐19 pandemic, such as anxiety about damages to the healthcare system or fear of infection, have previously been linked to maladaptive coping strategies (Taylor et al., 2020). We also identified that, compared to participants in paid employment, those not in paid employment were at greater risk of experiencing all outcomes. Financial worries and stress — that may arise due to a lack of paid employment — have been associated with mental health challenges during the pandemic (Flaudias et al., 2020; Herle et al., 2021; Paul & Fancourt, 2021; Sáiz et al., 2020; Sueki & Ueda, 2021; Wilson et al., 2020), and thus may explain our finding. Additionally, a sense of purpose has been reported to buffer against the impact of difficult experiences (Rutten et al., 2013). Having a definite purpose through paid employment may be particularly pertinent given that, during the pandemic, activities outside of work were severely limited. Consistent with our hypothesis and previous research (Elbogen et al., 2021; Iob et al., 2020; O'Connor et al., 2020; Sapara et al., 2021; Sueki & Ueda, 2021), being younger than mid‐life, specifically aged 16–25 years, was linked with higher odds of new‐onset low weight, suicidal and/or self‐harm ideation, and self‐harm. The pandemic exacerbated some known triggers for adverse mental health amongst young people, including conflict at home (Thompson et al., 2021), financial problems (Thompson et al., 2021), and social isolation (Hall‐Lande et al., 2007; McKinlay et al., 2022; Smith & Lim, 2020). Similarly to the latter finding, the related, although distinct (de Jong Gierveld et al., 2006), construct of self‐reported pandemic‐related loneliness in our study was linked to higher odds of suicidal and/or self‐harm ideation and binge eating.

Other shared risk factors included being a member of a vulnerable group, which was a risk factor for all outcomes. The pandemic is likely to have been a particularly stressful time for people vulnerable to infection, for instance, they may have experienced increased pressure to socially isolate, which may explain this finding. Consistent with our hypothesis and with previous research (Flaudias et al., 2020; Robertson et al., 2021), being female was a risk factor for new‐onset binge eating. In addition, being female was a risk factor for new‐onset suicidal and/or self‐harm ideation and self‐harm. However, contrary to our hypothesis and previous research (Flaudias et al., 2020; Herle et al., 2021), being female was not a risk factor for low weight. Our measure of low weight was binary and did not include any precise weight guidelines and is therefore open to subjective interpretation, which may explain our unexpected finding.

Another shared risk factor was being racially minoritized, which was associated with higher odds of new‐onset binge eating, low weight, and suicidal and/or self‐harm ideation during the pandemic. This is consistent with previous research that suggested frequent experiences of racial discrimination and having a weaker sense of British national identity — which may translate to feeling excluded from activities aimed to boost collective morale — are associated with greater fear of COVID‐19 (Jaspal & Lopes, 2021). This in turn is linked to adverse mental health consequences (Jaspal & Lopes, 2021). Indeed, the COVID‐19 pandemic has been linked with increased hate crimes and prejudice towards racially minoritized individuals, particularly Chinese people (Gray & Hansen, 2021; Roberto et al., 2020). Furthermore, the COVID‐19 pandemic has widened health inequalities; disproportionate numbers of infections and deaths have been reported in racially minoritized groups (Bhala et al., 2020; Yaya et al., 2020).

In addition to the shared risk factors outlined above, we also identified shared protective factors. For instance, contrary to our hypothesis, being infected with SARS‐CoV‐2/illness with COVID‐19 offered a protective effect against binge eating, low weight, and suicidal and/or self‐harm ideation. Most infections are mild (World Health Organization, 2022) or asymptomatic (Sah et al., 2021), and following recovery from such illness, participants may have felt relieved that the illness was not as severe as anticipated and/or that they now believed themselves to have some level of increased immunity. The potential for a subsequent reduction in anxiety or stress may explain our unexpected finding. Furthermore, participants who reported a change in their living situation had lower odds of new‐onset binge eating and suicidal and/or self‐harm ideation. This highlights that the ability to choose and adapt to changes in your environment may be important for your mental health, particularly at times of high stress. Indeed, living in a more crowded household or one without access to a garden has been linked to poorer mental health during the pandemic (Groot et al., 2022). However, it is important to note that choosing one's living situation is limited to a privileged minority; for most people, this is limited by financial constraints, place of work, and familial responsibilities. Thus, this finding may also reflect economic advantages.

Some risk and protective factors were specific to self‐harm symptoms. For example, being a minoritized gender was a risk factor for both self‐harm symptoms. Transgender people may have experienced particular difficulties in accessing hormone interventions, given that healthcare systems canceled or postponed elective procedures during the pandemic (Wang et al., 2020). Furthermore, non‐binary and transgender people experience misgendering and transphobia in healthcare (Carlile, 2020; Dolan et al., 2020; Whitehead, 2017), which may translate to healthcare avoidance, particularly during the COVID‐19 pandemic when healthcare visits were discouraged unless urgent (Tami et al., 2022). Contrary to our hypothesis, loss of a loved one or relative due to COVID‐19 was associated with lower odds of new‐onset suicidal and/or self‐harm ideation, despite the fact that people were often unable to visit their loved ones in hospital or attend funerals during the pandemic (Eisma et al., 2021; Eisma & Tamminga, 2020). Our finding may reflect a particular grief response in which, in anticipation of grief, people engage in proactive goal setting, live with greater intention, and re‐prioritize (Rogalla, 2020). Such behaviors may reduce the likelihood of experiencing new psychiatric symptoms. In addition, death from COVID‐19 is often preceded by a course of worsening illness that is distressing for loved ones. The death of someone suffering in this way may have offered some ease from stress for their loved ones. However, we would like to emphasize that our findings should not minimize the intensely difficult experience of losing a loved one during the pandemic, which some of the authors of this study directly experienced.

We also identified risk factors specific to eating disorder symptoms. For example, being a key worker was linked to higher odds of new‐onset low weight and binge eating. Previous research has suggested that key workers are vulnerable to adverse mental health because of challenges such as increased workload, as well as fear of contracting COVID‐19 and of infecting their loved ones (May et al., 2021). Indeed, both SARS‐CoV‐2 infection and subsequent COVID‐19 rates were higher in key workers than non‐key workers (EMG—Transmission Group, 2021; Topriceanu et al., 2021).

Our findings should be interpreted in light of limitations. While sample size is a key strength of our study, some of the eating disorder and self‐harm symptoms occurred rarely in individuals with certain exposures. For example, only 46 participants who reported a SARS‐CoV‐2 infection or illness with COVID‐19 also reported a new experience of suicidal and/or self‐harm ideation. Furthermore, our sample consisted of mostly white, cisgender, university‐educated, female participants, recruited to various volunteer cohorts, which may limit the generalizability of our findings to the wider population. Future recruitment efforts should focus on attracting those who are gender diverse, of color, and those who did not attend university. Third, some outcome measures were limited. The question about low weight was limited in its phrasing. For instance, we were unable to capture the important experiences of participants who may have engaged in restrictive eating and/or compensatory behaviors but not experienced low weight (i.e., atypical anorexia nervosa). Furthermore, the question about binge eating did not include a direct assessment of loss of control, a key aspect of binge eating (American Psychiatric Association, 2013; Sonneville et al., 2013). Future research should investigate the full spectrum of eating disorder symptoms (for instance, purging behaviors).

Overall, we detected subgroups, such as those with a psychiatric disorder, in a racially minoritized group, or not in paid employment, that were more likely to develop new onset of eating disorder or self‐harm symptoms during the pandemic. Close monitoring of people with these risk factors during future pandemics may enable early identification of new psychiatric symptoms.

## AUTHOR CONTRIBUTIONS

**Helena Lucy Davies:** Conceptualization; data curation; formal analysis; investigation; methodology; software; visualization; writing – original draft; writing – review and editing. **Christopher Hübel:** Conceptualization; data curation; formal analysis; investigation; methodology; project administration; software; supervision; writing – review and editing. **Moritz Herle:** Conceptualization; formal analysis; investigation; methodology; supervision; writing – review and editing. **Saakshi Kakar:** Data curation; formal analysis; software; validation; writing – review and editing. **Jessica Mundy:** Data curation; software; writing – review and editing. **Alicia J Peel:** Data curation; software; writing – review and editing. **Abigail R ter Kuile:** Data curation; software; writing – review and editing. **Johan Zvrskovec:** Data curation; software; writing – review and editing. **Dina Monssen:** Data curation; resources; writing – review and editing. **Kai Xiang Lim:** Writing – review and editing. **Molly R Davies:** Data curation; project administration; resources; writing – review and editing. **Alish B Palmos:** Data curation; software; writing – review and editing. **Yuhao Lin:** Data curation; software; writing – review and editing. **Gursharan Kalsi:** Data curation; funding acquisition; project administration; resources; writing – review and editing. **Henry C Rogers:** Data curation; resources; writing – review and editing. **Shannon Bristow:** Data curation; project administration; resources; writing – review and editing. **Kiran Glen:** Data curation; resources; writing – review and editing. **Chelsea Mika Malouf:** Data curation; resources; writing – review and editing. **Emily J Kelly:** Data curation; resources; writing – review and editing. **Kirstin Purves:** Data curation; funding acquisition; project administration; resources; writing – review and editing. **Katherine S Young:** Data curation; funding acquisition; project administration; resources; writing – review and editing. **Matthew Hotopf:** Funding acquisition; writing – review and editing. **Cherie Armour:** Funding acquisition; writing – review and editing. **Andrew M McIntosh:** Funding acquisition; writing – review and editing. **Thalia Eley:** Funding acquisition; writing – review and editing. **Janet Treasure:** Funding acquisition; project administration; supervision; writing – review and editing. **Gerome Breen:** Conceptualization; funding acquisition; investigation; methodology; project administration; supervision; writing – review and editing.

## FUNDING INFORMATION

This work was supported by the National Institute for Health and Care Research (NIHR) BioResource (RG94028, RG85445), NIHR Biomedical Research Centre (IS‐BRC‐1215‐20018), HSC R&D Division, Public Health Agency (COM/5516/18), MRC Mental Health Data Pathfinder Award (MC_PC_17,217), and the National Centre for Mental Health funding through Health and Care Research Wales. The RAMP study is supported in part by the King's Together Multi and Interdisciplinary Research Scheme (Rapid COVID‐19 call, Round 1; Round 2). Helena Davies and Alicia Peel acknowledge funding from the Economic and Social Research Council (ESRC) as part of a PhD studentship. Dr Hübel acknowledges funding from Lundbeckfonden (R276‐2018‐4581). Dr Herle is funded by a fellowship from the Medical Research Council UK (MR/T027843/1). Jessica Mundy acknowledges funding from the Lord Leverhulme Charitable Grant. Kai Xiang Lim acknowledges funding from King's International Postgraduate Research Scholarship. The RAMP study is supported in part by the King's Together Multi and Interdisciplinary Research Scheme (Rapid COVID‐19 call, Round 1; Round 2). Dr Young acknowledges funding from MQ; Transforming Mental Health (MQF20/24). Johan Zvrskovec and Abigail ter Kuile acknowledge funding from the National Institute for Health and Care Research (NIHR) Biomedical Research Centre and Guy's and St Thomas' NHS Foundation Trust.

## CONFLICT OF INTEREST

Prof Breen has received honoraria, research or conference grants and consulting fees from Illumina, Otsuka, and COMPASS Pathfinder Ltd. Prof Hotopf is principal investigator of the RADAR‐CNS consortium, an IMI public private partnership, and as such receives research funding from Janssen, UCB, Biogen, Lundbeck and MSD. Prof McIntosh has received research support from Eli Lilly, Janssen, and the Sackler Foundation, and has also received speaker fees from Illumina and Janssen.

## Supporting information


**Appendix S1:** Supporting Information

**Appendix S2:** Supporting Information

**Appendix S3:** Supporting Information

## Data Availability

The COPING study data are not publicly available however are available via a data request application to the NIHR BioResource (https://bioresource.nihr.ac.uk/using-our-bioresource/academic-and-clinical-researchers/apply-for-bioresource-data/). Deidentified RAMP Study data included in analyses presented here are available from study authors on request. All code for this study is publicly available: https://github.com/RAMP-COPING/EDBehaviour_SelfHarm.
