# Supplementary material for: Risk and protective factors for new‐onset binge eating, low weight, and self‐harm symptoms in >35,000 individuals in the UK during the COVID‐19 pandemic
Source: Int J Eat Disord. 2022 Oct 31;56(1):91–107. doi: 10.1002/eat.23834 (PMC9874817; doi:10.1002/eat.23834)
Supplement: Supplementary file 1 — Appendix S1: Supporting Information [file EAT-56--s001.docx]

**Supplementary materials**

**Risk and protective factors for new onset binge eating,** **low weight, and self-harm symptoms in >35,000 individuals in the UK during the COVID-19 pandemic**

Helena L. Davies^1(0000-0002-9419-1009)^, Christopher Hübel^1,2(0000-0002-1267-8287)^, Moritz Herle^1,3(0000-0003-3220-5070)^, Saakshi Kakar^1,2(0000-0003-1677-1857)^, Jessica Mundy^1(0000-0001-5513-8902)^, Alicia J. Peel^1(0000-0002-6144-5412)^, Abigail R. ter Kuile^1,2(0000-0002-7869-3754)^, Johan Zvrskovec^1,2(0000-0002-8862-0874)^, Dina Monssen^(0000-0003-0080-0799)1,2^, Kai Xiang Lim^(0000-0001-5709-4966)1^, Molly R. Davies^1,2(0000-0003-3483-9907)^, Alish B. Palmos^1,2(0000-0001-5748-6652)^, Yuhao Lin^1^, Gursharan Kalsi^(0000-0002-5156-7176)1,2^, Henry C. Rogers^1,2(0000-0003-2531-7496)^, Shannon Bristow^1,2(0000-0002-0896-781X)^, Kiran Glen^1,2(0000-0002-2831-3266)^, Chelsea Mika Malouf^1,2(0000-0002-5564-7464)^, Emily J Kelly^1,2(0000-0002-0118-4994)^, Kirstin L Purves^1(0000-0002-8110-5554)^, Katherine S. Young^1,2(0000-0002-1378-6415)^, Matthew Hotopf^4,2(0000-0002-3980-4466)^, Cherie Armour^5(0000-0001-7649-3874)^, Andrew M. McIntosh^6(0000-0002-0198-4588)^, Thalia C. Eley^1,2(0000-0001-6458-0700)^, Janet Treasure^7(0000-0003-0871-4596)^, Gerome Breen^1,2 (0000-0003-2053-1792)^

1. Social, Genetic and Developmental Psychiatry Centre, Institute of Psychiatry, Psychology and Neuroscience, King’s College London, London, UK
2. UK National Institute for Health and Care Research (NIHR) Biomedical Research Centre, South London and Maudsley Hospital, London, UK
3. Department of Biostatistics and Health Informatics, Institute of Psychiatry, Psychology & Neuroscience, King's College London, London, UK
4. Department of Psychological Medicine, Institute of Psychiatry, Psychology and Neuroscience, King’s College London, London, UK
5. Research Centre for Stress, Trauma and Related Conditions (STARC), School of Psychology, Queen's University Belfast, Belfast, Northern Ireland, UK
6. Division of Psychiatry, Centre for Clinical Brain Sciences, University of Edinburgh, Edinburgh, UK
7. Section of Eating Disorders, Department of Psychological Medicine, Institute of Psychiatry, Psychology and Neuroscience, King's College London, London, UK

**1 SUPPLEMENTARY METHODS**

**1.1 Exposures in main analysis**

We measured a range of demographic, clinical, and COVID-related exposure variables at baseline assessment. Below, we have described how we defined each of them for this study.

**Age.** Age was assessed in the COPING study and RAMP Study baseline surveys, in the 'demographics' questionnaire. In the RAMP Study, participants were asked their age category. Therefore, we collapsed age from all other cohorts into categories (i.e., 16-25, 26-35, 46-55, 56-65, 66-70, and 71+ years).

**Sex.** All NIHR BioResource participants were asked their assigned sex at birth in the COPING study baseline survey, in the 'demographics' questionnaire (i.e., *'Male'* or *'Female'*). RAMP Study participants were not explicitly asked their sex. Thus, for RAMP Study participants, we used their self-reported gender and whether they considered themselves transgender to decipher their probable assigned sex at birth.

**Minoritised gender.** Gender was self-reported in the COPING study and RAMP Study baseline surveys, again in the 'demographics' questionnaire, with options of *'Male'*, *'Female'*, '*Non-binary*' and '*Prefer to self-define*'. We categorised responses of *'Male'* as 'being a man' and *'Female'* as 'being a woman'. Responses of '*Non-binary*' and *'Prefer to self-define*' were categorised as ‘minoritised gender’. In the GLAD Study and EDGI UK sign-up surveys and the RAMP Study baseline survey, participants were also asked whether they identify as transgender. Responses of transgender were additionally classified as 'minoritised gender'. Sample sizes in these more refined groups within the ‘minoritised gender’ category were too small to keep them as independent categories.

**Racially minoritised.** Race was self-reported in the GLAD Study and EDGI UK sign-up 'demographic' questionnaires. Answer options included: *'White'*, *'Mixed'*, '*Asian or Asian British*', '*Black or Black British*', *'Arab'*, and *'Other'*. Other NIHR BioResource cohorts self-reported their race at initial enrolment to the BioResource (e.g., '*Asian or Asian British - Indian*', '*Black or Black British - Caribbean*'). RAMP Study participants were asked their race in the 'demographics' questionnaire in the RAMP Study baseline survey (e.g., '*White, white European or Caucasian*', '*Black or Black British*'). For participants who self-reported being white, we categorised them as 'not racially minoritised'. For all other participants, we categorised them as 'racially minoritised' (again, sample sizes in the more refined racial groups were too small to keep them as independent categories).

**Psychiatric disorder.** Participants who indicated having any psychiatric disorder diagnosis in their respective sign-up or baseline surveys were classified as having a psychiatric disorder. The answer options included: ‘*Major depressive disorder’, ‘Perinatal depression’, ‘Premenstrual dysphoric disorder’, Bipolar disorder’, ‘Generalised anxiety disorder’, ‘Social anxiety disorder’, ‘Specific phobia, agoraphobia’, ‘Panic disorder’, ‘Post-traumatic stress disorder’, ‘Obsessive compulsive disorder (OCD)’, ‘Body dysmorphic disorder’, ‘Other OCD’, ‘Anorexia nervosa*’, ‘Atypical anorexia nervosa’, ‘Bulimia nervosa*’, ‘Binge-eating disorder**’, *‘Atypical bulimia nervosa*’, ‘Atypical binge-eating disorder*’, ‘Purging disorder’, ‘Night eating syndrome’, ‘Pica’, ‘Avoidant Restrictive Food Intake Disorder’, ‘Rumination disorder’, ‘Other feeding or eating disorder’, ‘Schizophrenia’, ‘Schizoaffective disorder’, ‘Psychosis’, ‘Personality disorder’, ‘Autism spectrum disorder’,* and *‘Attention Deficit Hyperactivity Disorder*’. Additionally, responses of *'Yes'* to the question *'Have you ever received treatment for an eating disorder?*' were considered as endorsement of having a diagnosed eating disorder. In addition to self-reported diagnoses, we identified eating disorder cases (anorexia nervosa*, bulimia nervosa*, and binge-eating disorder*) using DSM-5 algorithms made up of responses to the ED100K[^2^](https://paperpile.com/c/zGXroz/y1Esp). Participants who answered ‘*None of the above*’ to all questions about diagnosed psychiatric disorders and who did not meet criteria for any of the algorithmically-derived eating disorders were classified as not having a psychiatric disorder at baseline.

*Note: As we outline in our definition of our outcomes in section 1.2, a large number of participants with eating disorders that include the symptoms of binge eating and/or low weight were identified as having pre-pandemic experience of the outcome and thus were dropped from further analyses.

**Paid employment and key worker status**

**Key worker.** In the COPING study and RAMP Study baseline survey, participants were asked, '*Do you work in any of these professions identified as key workers by the government?*'. Participants who endorsed any of the listed professions (*'Health and social care', 'Education and childcare', 'Key public services [i.e. justice system, journalists, religious staff, responsible for dealing with the deceased]', 'Key public services [i.e. refuse collection and maintenance, water, gas electricity, infrastructure]', 'Local and national government', 'Involved in production, processing, distribution or delivery of food or other necessary goods', 'Public safety and national security [including military and police personnel]', 'Transport', 'Utilities, communication and financial services',* or *'Other'*) were identified as key workers. Participants who answered '*None of these*' were identified as not being key workers.

**Paid employment.** In the COPING study and RAMP Study baseline surveys, participants were asked '*What was your employment status prior to the pandemic?*'. All participants who endorsed *'Retired'*, '*Student (GCSE or A level)*', or '*Student (University)*', were grouped into 'Retired' or 'Student', respectively. These participants were not given any follow-up employment questions. Participants who endorsed any other answer option were subsequently asked, *'Has your employment status changed since the pandemic began?*'. To decipher employment status at baseline, we considered participants’ answers to both questions. Any participant who indicated previously being in paid employment (i.e., *'Full-time employed', 'Part-time employed', 'Zero-hours contract', 'Self-employed', 'Contract or freelance work'* or *'Small business owner'*) and who then endorsed one of the following options: *'My employment status has not changed', 'Reduction in hours', 'Reduction in salary', 'Benefits increased', 'Benefits decreased', 'Change in duties or responsibilities', 'Increased hours', 'Increased salary', 'Furloughed or paid leave (Government funded)', 'Furloughed or paid leave (Company funded)', 'Furloughed or paid leave (Government funded with company supplement)'* or *'Other'*, were classified as being in paid employment at baseline assessment. Additionally, participants who endorsed the answer option of '*Became employed*' in the follow-up question were also classified as being in paid employment at baseline assessment.

Contrastingly, participants who indicated that previously they were not in paid employment, (i.e., *'Unemployed'*, '*Stay-at-home parent or carer*', '*Receiving state income*') and who subsequently endorsed any of the following options: '*My employment status has not changed', 'Benefits increased', 'Benefits decreased', 'Change in duties or responsibilities',* or *'Other'*, were categorised as not being in paid employment at baseline. Additionally, all NIHR BioResource participants were given the answer option of '*Taking unpaid leave*', which we categorised as not being in paid employment. Finally, all participants who answered '*Became unemployed*' to the follow-up question were identified as not being in paid employment. For participants who did not answer the follow-up question '*Has your employment status changed since the pandemic began?'*, we used their answers from the question ‘*What was your employment status prior to the pandemic?’* to decipher their employment status at baseline.

From the above, we grouped participants into the following categories: 1) in paid employment but not a key worker, 2) key worker (and therefore in paid employment), 3) not in paid employment, 4) student, and 5) retired.

**Pandemic worry.** In the COPING study and RAMP Study baseline survey, participants were asked their recent levels of worry about 21 different items, '*Over the past two weeks, how worried have you been about the following issues?*'. The listed issues included: *'Being socially isolated', 'People you know being socially isolated', 'Shortage of essential supplies', 'Shortage of medication or access to healthcare', 'Shortage of essential supplies or healthcare for people you know', 'Accuracy of information about the virus from the government and social media', 'The government's response to the pandemic', 'Separation from family members', 'A global recession or long-standing impact on the economy', 'Impact on your employment status', 'The employment status of other key earners in your household', 'Impact on your education or exams', 'Impact on the education or exams of your children', 'Financial impact', 'Contracting the virus', 'People you know contracting the virus', 'People you don't know contracting the virus', 'Impact on your own mental health and wellbeing', 'Impact on your children’s mental health and wellbeing', 'Impact on the mental health of other relatives',* and *'Other'.* We first calculated the missingness across each item. The item *'Other'* had higher missingness (36.2%) than all other listed items (11.3-11.6%). Therefore, we dropped this item from the overall sum score. Answer options and their assigned scores were: '*Not applicable*' (0), '*Not at all worried*' (0), '*Not too worried*' (1), '*Somewhat worried*' (2), '*Very worried*' (3), '*Extremely worried*' (4). We calculated sum scores with complete cases, in which the minimum score was 0 and the maximum was 80. We grouped participants into scores of 0-20, 21-40, 41-60, and 61-80.

**Member of a vulnerable group.** Participants who answered *'Yes'* to the question, '*Are you a member of a vulnerable group in need of additional isolation or protective measures during the pandemic as identified by the government?*' in the COPING study and RAMP Study baseline survey were considered members of a vulnerable group. Those who answered *'No'* were classified as not being part of a vulnerable group.

**Pandemic loneliness.** In the COPING study and RAMP Study baseline surveys, participants were asked: '*Over the past two weeks, how often have you felt that the following statements apply to you?*'. The following statements were: '*Felt that you lack companionship*', '*Felt left out'*, '*​​Felt isolated from others*', and '*Felt alone*'. The answer options for each statement were: *‘Hardly ever*’, ‘*Some of the time’*, and *‘Often’*.

Participants were then asked ‘*How similar is this to how often you felt this way before the pandemic?*’ with the answer options of *‘I felt this way much more frequently’*, ‘*I felt this way a little more frequently’ ‘No different’, ‘I felt this way a little less frequently’,* and *‘I felt this way much less frequently’.* Those who indicated a change (i.e., answered anything other than ‘*No different*’) were then asked '*Before the pandemic, how often have you felt that the following statements apply to you?*' with the same statements. Again, the answer options were: ‘*Hardly ever’*, ‘*Some of the time*’, and *‘Often’*.

We calculated a 'retrospective pre-pandemic loneliness score' and 'pandemic loneliness score', in which, to each relevant question, answers of '*Hardly ever*' were scored as 0, '*Some of the time*' were 1, and *'Often'* were 2. The minimum possible score for each was 0 and the maximum was 8. People whose ‘pandemic loneliness scores’ were higher than their ‘retrospective pre-pandemic loneliness scores’ were categorised as experiencing higher loneliness during pandemic. People whose ‘pandemic loneliness scores’ were the same as or lower than their ‘retrospective pre-pandemic scores’ were categorised as having not experienced greater loneliness during the pandemic. Participants who did not get asked about the follow-up question concerning pre-pandemic loneliness because they indicated that it was ‘*No different*’ to their loneliness during the pandemic were also categorised as having not experienced greater loneliness during the pandemic.

**Exposure before outcome**

For all of the below exposures, we utilised data from each follow-up phase as well as data from the baseline survey (if available). If a participant self-reported experiencing both the exposure and the outcome, their data were only included in the relevant regression model if their first self-report of the exposure occurred in the same phase or before the phase in which they first self-reported the outcome. For participants who answered twice within a single phase (i.e., answered one survey late and the next on time), we counted an answer of *'Yes'* to the outcome or exposure in either response during the phase as an endorsement during that phase. For the remaining participants, we counted an answer of *'No'* to the outcome or exposure in either phase as the participants not having experienced the relevant outcome or exposure during that phase.

**COVID-19 infection.** We assessed whether a participant had been infected with COVID-19 via questions included in three questionnaires. First, in every other COPING study and RAMP Study follow-up survey, participants were given a 'virus' questionnaire, which asked: '*Have you ever had a COVID-19 test? Please include any tests you’ve reported in past surveys'* and *'Have any of your tests come back positive for COVID-19 or antibodies?*'. Second, a 'respiratory' questionnaire, which was included at baseline and at every follow up phase, asked: '*Did you have a nose/throat swab to test for Coronavirus in the last week?'* and '*What were the results of the nose/throat swab test?*' and '*Did you have an antibody test for Coronavirus in the last week?*' and '*What were the results of the antibody test?*'. Third, in the 'demographics' questionnaire included in every follow-up phase, participants were asked, '*In the last two weeks, has your physical health changed because you became unwell with the coronavirus?*'.

Participants were categorised as having been infected with COVID-19 if, at any point, they answered *'Positive'* to any of the three questions in the 'virus' and 'respiratory' questionnaires about the test results or answered *'Yes'* to the question about being unwell with the coronavirus in the 'demographics' questionnaire. Participants who never answered any of the aforementioned questions at any phase or who indicated that they had taken a test but did not indicate the test results were labelled as having missing data for this variable. Out of the remaining participants, those who indicated at least once during the monitoring period that they had not taken a test, that their results were negative, and/or that their physical health had not changed because of COVID-19 illness were classified as having not been ill with COVID-19.

**Loss of loved one or relative due to COVID-19.** Two questionnaires included in the COPING study and RAMP Study surveys enabled assessment of this variable. First, the 'grief' questionnaire, which was given to participants at baseline and at every follow-up survey, and asked participants: '*In the past month, have you lost someone close to you due to COVID-19?'.* Second, the question '*Has a relative that was previously ill with coronavirus or suspected coronavirus passed away?'* was included in the 'demographics' questionnaire and was given to participants at every follow-up.

Participants who at any point answered *'Yes'* to either question were categorised as having lost someone due to COVID-19. Participants who never answered or who only ever answered '*Prefer not to say*' were labelled as having missing data for this variable. Of the remaining participants, those who answered *'No'* at least once to either question were categorised as not having lost someone due to COVID.

**Change in main economic activity: Employment.** In the COPING study and RAMP Study baseline 'employment' questionnaire, participants were asked: '*Has your employment status changed since the pandemic began?'* and given the answer options of: *'My employment status has not changed', 'Became unemployed', 'Reduction in hours', 'Reduction in salary', 'Benefits increased', 'Benefits decreased', 'Change in duties or responsibilities', 'Became employed', 'Increased hours', 'Increased salary', 'Furloughed or paid leave (Government funded)', 'Furloughed or paid leave (Company funded)', 'Furloughed or paid leave (Government funded with company supplement)', 'Taking unpaid leave',* or *'Other'.* Then, at every follow-up phase, participants were asked: *'Has anything changed in the following domains in the last two weeks?'* with the following answer options: *'Nothing has changed', 'Living situation', 'Employment', 'Physical health', 'Health of a relative', 'Employment status of a key earner in your household',* and *'Children/dependants returning to school or other education'.*

To the first question, participants who indicated anything other than '*My employment status has not changed'* at baseline were identified as having experienced a change in their main economic activity at baseline. People who answered '*My employment status has not changed*' were identified as having not experienced a change in their main economic activity at baseline. Similarly, participants who, at any phase, endorsed *'Employment*' or '*Employment status of a key earner in your household*' were categorised as having experienced a change in their main economic activity during the pandemic. People who indicated that they have not experienced a change in their main economic activity (i.e., by answering the question but not endorsing *'Employment'* or '*Employment status of a key earner in your household'*) at least once were categorised as having not experienced a change in their main economic activity.

**Change in main economic activity: Education.** Participants who had previously indicated they were a student were asked (regarding the COVID-19 pandemic), *'How has this impacted your studies?*' in the COPING study and RAMP Study baseline employment questionnaire. Answer options included *'No impact', 'Cancelled exams', 'Change in format of exams', 'Automatic pass', 'Change in deadlines', 'Change in lesson delivery', 'Grade decided based on past performance (e.g. coursework, predicted grades)', 'Cancelled classes', 'Cannot complete degree', 'Impact on studies still unknown',* or *'Other'.* Participants who answered anything other than ‘*No impact’* and '*Impact on studies still unknown*' were counted as having experienced a change in their main economic activity. Participants who indicated '*No impact'* or *'Impact on studies still unknown'* were categorised as having not experienced a change in their main economic activity. This question was only included at baseline.

**Change in living situation.** In the COPING study and RAMP Study baseline 'demographics' questionnaire, participants were asked: '*What is your living status right now, during the pandemic?*', followed by: *'Is this a change from your living situation before the pandemic?*'. Participants who answered *'Yes'* to the latter question were identified as having experienced a change of living situation at baseline. Responses to the aforementioned question, '*Has anything changed in the following domains in the last two weeks?*', were used to establish whether participants had experienced a change in living situation in any of the follow-up phases (one answer option was '*Living situation*'). Participants who, at any phase, endorsed *'Living situation*' were categorised as having experienced a change in their living situation during the pandemic. People who indicated that they had not experienced a change in their living situation (i.e., by answering the question but not selecting '*Living situation*') at least once were categorised as not having experienced a change in their living situation.

**1.2 Outcomes**

At frequent intervals, participants reported their experience of binge eating, low weight, passive suicidal ideation, self-harm ideation, and self-harm. Pre-pandemic experiences of each outcome - which in turn informed whether an experience during the pandemic was ‘new’ - were derived differently within and across each dataset due to variations in the available data. As outlined below, we wanted to first identify people without pre-pandemic experience of the outcomes. These participants form the basis of our analysis. We then identify who went on to newly experience each outcome during the pandemic.

**1.2.1 No pre-pandemic experience of low weight**

**EDGI UK.** EDGI UK survey participants were not asked questions about eating disorder symptoms in the COPING study baseline survey (EDGI UK launched in February 2020 so we had already collected recent symptom information). Therefore, only answers to the EDGI UK sign-up survey were available to identify whether an EDGI UK survey participant had pre-pandemic experience of low weight. Participants who did not endorse a lifetime diagnosis of anorexia nervosa in the MHD questionnaire[^1^](https://paperpile.com/c/zGXroz/XpWsi) and did not report a lifetime experience of low weight in the ED100K[^2^](https://paperpile.com/c/zGXroz/y1Esp) screener were classified as not having pre-pandemic experience of low weight. We took answers to the low weight question in the ED100K[^2^](https://paperpile.com/c/zGXroz/y1Esp) for participants with missing diagnosis data, as it was the symptom of low weight that was our primary focus.

EDGI UK survey participants were asked about the age at which they experienced low weight. Therefore, for the participants who answered the EDGI UK sign-up survey during the pandemic and endorsed a lifetime experience of low weight, we were able to identify whether this was likely to be a newly-occurring pandemic experience or a pre-pandemic experience. We contrasted their age at symptom start with their age at the beginning of the pandemic. We dropped participants from our analysis who indicated that they were older or the same age at the start of the pandemic than they were when they first experienced low weight. Those who indicated that they were younger at the start of the pandemic than they were when they experienced low weight were classified as having a newly-occurring pandemic experience of low weight, and thus no pre-pandemic experience.

**GLAD Study.** GLAD Study survey participants in this study have answered both the GLAD Study survey (which includes the optional ED100K[^2^](https://paperpile.com/c/zGXroz/y1Esp)) and the COPING baseline study survey (which includes the mandatory ED100K[^2^](https://paperpile.com/c/zGXroz/y1Esp)). We have utilised responses to both surveys to capture those with no pre-pandemic experience of our eating disorder outcomes (low weight and binge eating).

GLAD Study survey participants were asked questions about anorexia nervosa in the GLAD Study sign-up survey via the optional ED100K[^2^](https://paperpile.com/c/zGXroz/y1Esp) and the MHD questionnaire[^1^](https://paperpile.com/c/zGXroz/XpWsi). To identify those without pre-pandemic experience of low weight, we looked at only those who had completed the optional ED100K[^2^](https://paperpile.com/c/zGXroz/y1Esp) or the MHD[^1^](https://paperpile.com/c/zGXroz/XpWsi) within the GLAD Study survey after the 23rd January 2020. We chose the 23rd January 2020 as it is three months before the start of the pandemic; we have assumed that if participants do not report low weight after this date, any experience reported during the pandemic is likely to be for the first time. GLAD survey participants who, after this date, indicated that they did not have a lifetime experience of low weight ('*Have you ever had a period of time when you weighed much less than other people thought you ought to weigh?*') or a lifetime diagnosis of anorexia nervosa were classified as not having pre-pandemic experience of low weight.

GLAD Study survey participants were asked for their age at symptom start, i.e., the age at which they first experienced low weight. Therefore, for GLAD Study survey participants who endorsed lifetime low weight and answered the GLAD Study survey during the pandemic, those with an age at low weight that was younger than or the same as their age at the start of the pandemic were dropped from further analyses, as they were categorised as having pre-pandemic experience of low weight. Participants whose age at symptom start was older than their age at the start of the pandemic were categorised as having a newly-occurring pandemic experience of low weight and therefore also categorised as having no pre-pandemic experience of low weight.

GLAD Study survey participants also filled out the COPING study baseline survey which contained questions about eating disorders, including age at symptom start. The COPING study survey was launched during the pandemic (April 2020). Therefore, those who reported no lifetime diagnosis of anorexia and no lifetime experience of low weight were categorised as having no pre-pandemic experience of low weight. Participants who endorsed the ED100K[^2^](https://paperpile.com/c/zGXroz/y1Esp) anorexia screener question in the COPING baseline survey, '*Have you ever had a period of time when you weighed much less than other people thought you ought to weigh?*' and who, to the question, '*How old were you then?'* indicated an age younger than or the same as their age at the start of the pandemic, were classified as likely to have pre-pandemic experience of low weight and were dropped from our analyses. Again, those with an age at symptom start older than their pandemic start age were classified as having a newly-occurring pandemic experience of low weight, and therefore no pre-pandemic experience.

GLAD Study survey participants who, in either the GLAD Study sign-up survey or the COPING study survey indicated pre-pandemic experience of low weight were dropped from our analyses. Those with no pre-pandemic experience of low weight were those who, of the remaining participants, were identified as having no pre-pandemic experience in either the GLAD Study survey and/or in the COPING study survey as outlined above. For participants with missing diagnosis data in both surveys, we took answers to the low weight question/s in the ED100K[^2^](https://paperpile.com/c/zGXroz/y1Esp).

**Remaining NIHR BioResource cohorts (IBD; COMPARE; STRIDES; INTERVAL; RTB-GEN).** The remaining NIHR participants were asked questions about low weight in the COPING study baseline survey, including age at symptom start. We identified participants without pre-pandemic experience of low weight in the same way as the GLAD survey participants who completed the COPING study survey, as outlined above.

**RAMP Study.** RAMP Study participants were only asked about diagnoses in the MHD questionnaire[^1^](https://paperpile.com/c/zGXroz/XpWsi) in the RAMP Study baseline survey. The participants were not given the ED100K[^2^](https://paperpile.com/c/zGXroz/y1Esp) so symptom data at baseline were not available. Thus, any RAMP Study participant who indicated that they had not received a lifetime diagnosis of anorexia nervosa were classified as not having pre-pandemic experience of low weight. Those who endorsed a lifetime diagnosis were dropped from our analyses.

**1.2.2 New-onset low weight during the pandemic**

In the RAMP Study baseline survey and in every other RAMP Study and COPING study follow-up survey, participants were asked, *'Over the past month, have you weighed much less than other people thought you ought to weigh?'*. Participants with no pre-pandemic experience of low weight (established as outlined above) and who, at any point during the monitoring period, endorsed an experience of low weight, were classified as having a new onset of low weight during the pandemic. Of the remaining participants without pre-pandemic experience of low weight, those who answered *'No'* at least once were classified as not experiencing new onset of low weight.

**1.2.3 No pre-pandemic experience of binge eating**

**EDGI UK.** An EDGI UK survey participant was categorised as not having a pre-pandemic experience of binge eating if, in the EDGI UK sign-up survey, they did not self-report a lifetime diagnosis of bulimia nervosa, binge-eating disorder, atypical binge-eating disorder, or atypical bulimia nervosa in the MHD[^1^](https://paperpile.com/c/zGXroz/XpWsi) and answered *'No'* to the question *'Have you ever had regular episodes of overeating or eating binges when you ate what most people would regard as an unusually large amount of food in a short period of time?'* in the ED100K[^2^](https://paperpile.com/c/zGXroz/y1Esp). Participants who answered the EDGI UK survey during the pandemic (i.e., on or after the 23rd March 2020) and endorsed a lifetime diagnosis of a binge-type eating disorder (as listed above) were categorised as having pre-pandemic experience of binge eating, given that diagnostic delays means it is likely symptoms started before the pandemic. For those who, during the pandemic, reported a lifetime experience of binge eating, we contrasted their age at symptom start with their age at the beginning of the pandemic. We then applied the same rules to binge eating and age at symptom start as we did to low weight (outlined above) to identify EDGI UK survey participants with no pre-pandemic experience of binge eating. For participants with missing diagnosis data, we took answers to the binge eating question in the ED100K[^2^](https://paperpile.com/c/zGXroz/y1Esp).

**GLAD Study.** To be categorised as not having pre-pandemic experience of binge eating, GLAD Study survey participants must have, in the GLAD Study sign-up survey after the 23rd January 2020, answered *'No'* to the question *'Have you ever had regular episodes of overeating or eating binges when you ate what most people would regard as an unusually large amount of food in a short period of time?'* in the optional ED100K[^2^](https://paperpile.com/c/zGXroz/y1Esp), and have not self-reported a diagnosis of a binge-type eating disorder in the MHD questionnaire[^1^](https://paperpile.com/c/zGXroz/XpWsi) or in the optional ED100K[^2^](https://paperpile.com/c/zGXroz/y1Esp). For those who answered the GLAD Study or COPING study survey during the pandemic and self-reported lifetime binge eating, we cross-checked their age at symptom start with their age at the start of the pandemic. We applied the same rules to binge eating as we did to low weight (outlined above) to identify GLAD Study survey participants with no pre-pandemic experience of binge eating.

**Remaining NIHR BioResource cohorts (IBD; COMPARE; STRIDES; INTERVAL; RTB-GEN).** The remaining NIHR BioResource participants were asked questions about binge eating in the COPING study baseline survey in the same way as the GLAD survey participants in the COPING survey. Therefore, we identified participants without pre-pandemic experience of binge eating in the same way as in the GLAD Study, as outlined above.

**RAMP Study.**  At baseline, RAMP Study participants were given a screener to the EDE-Q[^3^](https://paperpile.com/c/zGXroz/NpoM), *'Over the past month, have you had regular episodes of overeating or eating binges when you ate what most people would regard as an unusually large amount of food in a short period of time?'*. Because the RAMP Study started during the pandemic (April 2020)*,* the 'past month' in reference is during the pandemic. Therefore, an answer of *'Yes'* only indicated whether a participant had a pandemic experience of binge eating. However, those who answered *'Yes'* were subsequently directed to the EDE-Q, which included the question: *'Over the past 28 days BEFORE PANDEMIC, how many times have you: Eaten what other people would regard as an unusually large amount of food with a sense of having lost control over your eating?'.* Therefore, any RAMP Study participant who either did not get asked this question about binge eating before the pandemic (i.e., did not pass the EDE-Q screener question) or who gave an answer of zero, and who also self-reported that they did not have a lifetime diagnosis of '*Psychological overeating or binge-eating disorder'* or '*Bulimia nervosa*' were classified as not having pre-pandemic experience of binge eating.

**1.2.4 New-onset binge eating during the pandemic**

**All cohorts.** In the RAMP Study baseline survey and in every other RAMP Study and COPING study follow-up survey, participants were asked, '*Over the past month, have you had regular episodes of overeating or eating binges when you ate what most people would regard as an unusually large amount of food in a short period of time?'*. Participants who had been identified as having no pre-pandemic experience of binge eating (established as outlined above) and who, at any point during the monitoring period, endorsed an experience of binge eating, were classified as having new onset binge eating. Of the remaining participants without pre-pandemic experience of binge eating, those who answered *'No'* at least once were classified as not newly experiencing binge eating during the pandemic.

**1.2.5 No pre-pandemic experience of passive suicidal ideation**

**All cohorts.** In the COPING study and RAMP Study baseline surveys, participants were asked: *'Many people have thoughts that life is not worth living. Have you felt that way?'*. Participants who answered *‘No’* were classified as having no pre-pandemic experience of passive suicidal ideation. Participants who answered *'Yes, once'* or *'Yes, more than once'* were then asked *'Had you felt that way before the pandemic?*'. Of these participants, those who answered *'No'* to this question were classified as having new-onset passive suicidal ideation during the pandemic and thus also no pre-pandemic experience.

**1.2.6 New-onset passive suicidal ideation during the pandemic**

**All cohorts.** At every follow-up phase, participants were asked the following questions: *'Many people have thoughts that life is not worth living. Have you felt that way?’* and *‘Have you felt that way in the past two weeks?’.* Those with no pre-pandemic experience and who, at any point during the monitoring period (including at baseline) endorsed either of these questions, were classified as having new-onset passive suicidal ideation. Of the remaining participants without pre-pandemic experience of passive suicidal ideation, those who answered *'No'* at least once were classified as not having newly experienced passive suicidal ideation during the pandemic.

**1.2.7 No pre-pandemic experience of self-harm ideation**

**All cohorts.** In the COPING study and RAMP Study baseline surveys, participants were asked: *'Have you contemplated harming yourself?'.* Participants who answered *‘No’* were classified as having no pre-pandemic experience of self-harm ideation. Participants who answered *'Yes, once'* or *'Yes, more than once'* were then asked *'Had you felt that way before the pandemic?'.* Of these participants, those who answered *'No'* to this question were classified as having new-onset passive suicidal ideation during the pandemic and thus also no pre-pandemic experience.

**1.2.8 New onset self-harm ideation during the pandemic**

**All cohorts.** At every follow-up phase, participants were asked the following questions: *'Have you contemplated harming yourself?’* and *‘Have you felt that way in the past two weeks?’.* Those with no pre-pandemic experience and who, at any point during the monitoring period (including at baseline) endorsed either of these questions, were classified as having new onset self-harm ideation. Of the remaining participants without pre-pandemic experience of self-harm ideation, those who answered *'No'* at least once were classified as not having newly experienced self-harm ideation during the pandemic.

**1.2.9 No pre-pandemic experience of self-harm**

**All cohorts.** In the COPING study and RAMP Study baseline surveys, participants were asked: *'Before the pandemic, had you deliberately harmed yourself, whether or not you meant to end your life?'*. Participants who answered *'No'* were categorised as not having pre-pandemic experience of self-harm.

**1.2.10 New onset self-harm during the pandemic**

**All cohorts.** At baseline and in every follow-up phase, participants were also asked, *'In the last two weeks, have you deliberately harmed yourself, whether or not you meant to end your life?*'. Those with no pre-pandemic experience and who, at any point during the monitoring period (including at baseline) endorsed self-harm, were categorised as having new-onset self-harm. Of the remaining participants without pre-pandemic experience of self-harm, those who answered *‘No’* at least once were classified as not having newly experienced self-harm during the pandemic.


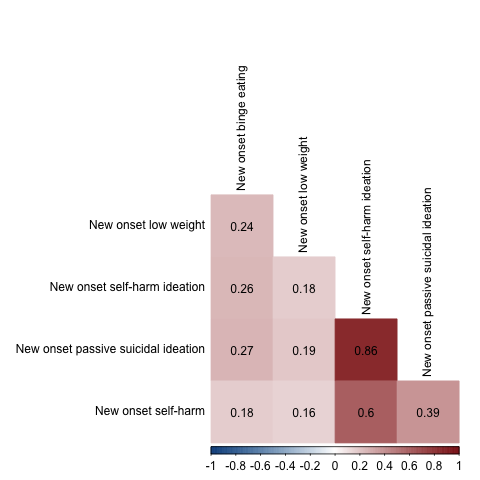


**SUPPLEMENTARY FIGURE 1** Correlation matrix for all outcomes: binge eating, low weight, self-harm ideation, passive suicidal ideation, and self-harm, in the analysis sample (*n* = 36,715). All correlations are tetrachoric correlations.

**SUPPLEMENTARY TABLE 1** Characteristics of the participants included in the main analysis (*n* = 36,715) versus participants excluded from analyses (*n* = 8,356) due to missing data.

|  | Included participants | Excluded participants | Significance of difference (p value) |
| --- | --- | --- | --- |
| *n* | 36,715 | 8,356 |  |
| Age: 16-25 years | 3,184 (8.67%) | 2,102 (25.17%) | 2.16x10^-16^ |
| Age: 26-35 years | 4,867 (13.26%) | 1,978 (23.68%) | 7.94x10^-127^ |
| Age: 36-45 years | 5,005 (13.63%) | 1,362 (16.31%) | 2.45x10^-10^ |
| Age: 46-55 years | 7,559 (20.58%) | 1,378 (16.50%) | 2.53x10^-17^ |
| Age: 56-65 years | 9,057 (24.67%) | 981 (11.74%) | 7.96x10^-145^ |
| Age: 66-70 years | 3,640 (9.91%) | 291 (3.48%) | 7.45x10^-79^ |
| Age: 71+ years | 3,401 (9.26%) | 261 (3.13%) | 1.09x10^-76^ |
| Female | 25,728 (70.29%) | 6,709 (80.70%) | 1.78x10^-81^ |
| Racially minoritised | 1,348 (3.75%) | 523 (6.40%) | 5.38x10^-27^ |
| Minoritised gender | 516 (1.41%) | 327 (3.93%) | 6.22x10^-53^ |
| AS levels or higher | 29,462 (80.25%) | 6,065 (72.58%) | 1.18x10^-12^ |
| BMI at registration [kg/m2] (median, IQR) | 29.0 (8.3) | 28.70 (10.24) | 3.40x10^-4^ |

Note. Minoritised gender = ‘Transgender’, ‘Non-binary’, and ‘Prefer to self-define’; Racially minoritised = ‘Arab’, ‘Asian’, ‘Black’, ‘Mixed race’, and ‘Other’; IQR =  interquartile range. Percentages are based on complete data, therefore may not reflect the numbers in the table.


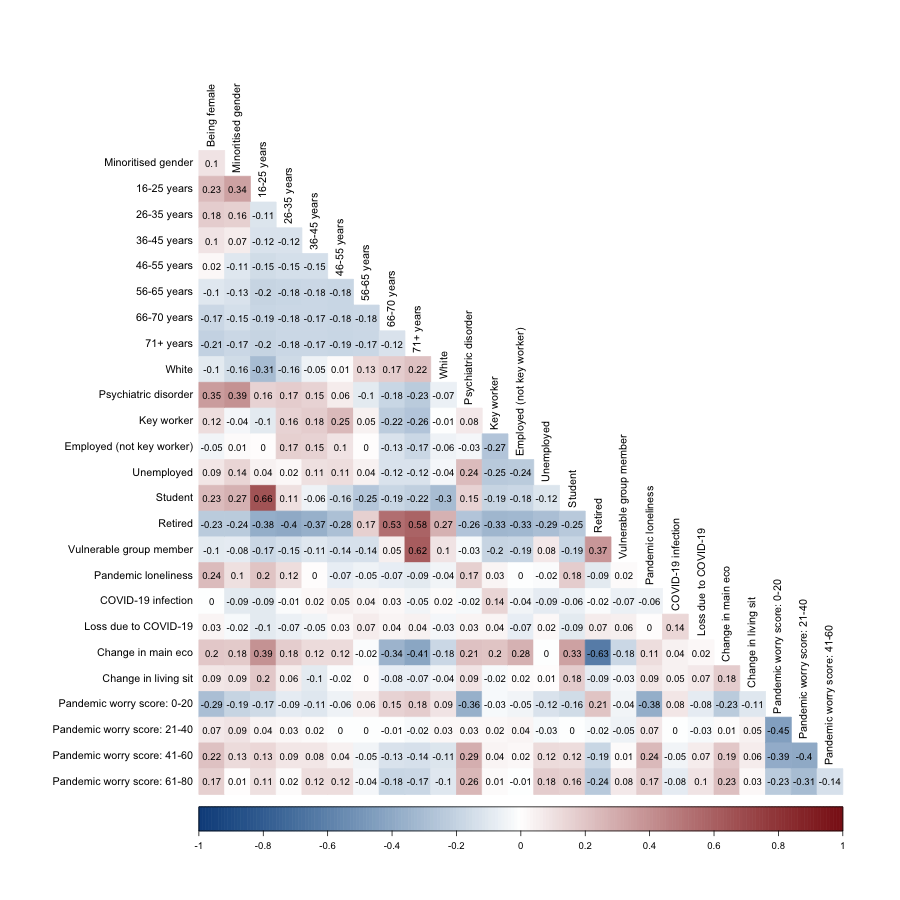


**SUPPLEMENTARY FIGURE 2** Correlation matrix for all exposures included in the regression model with the outcome binge eating (*n* = 24,211). All correlations are tetrachoric correlations. Note. Minoritised gender = ‘Transgender’, ‘Non-binary’, and ‘Prefer to self-define’; Racially minoritised = ‘Arab’, ‘Asian’, ‘Black’, ‘Mixed race’, and ‘Other’.


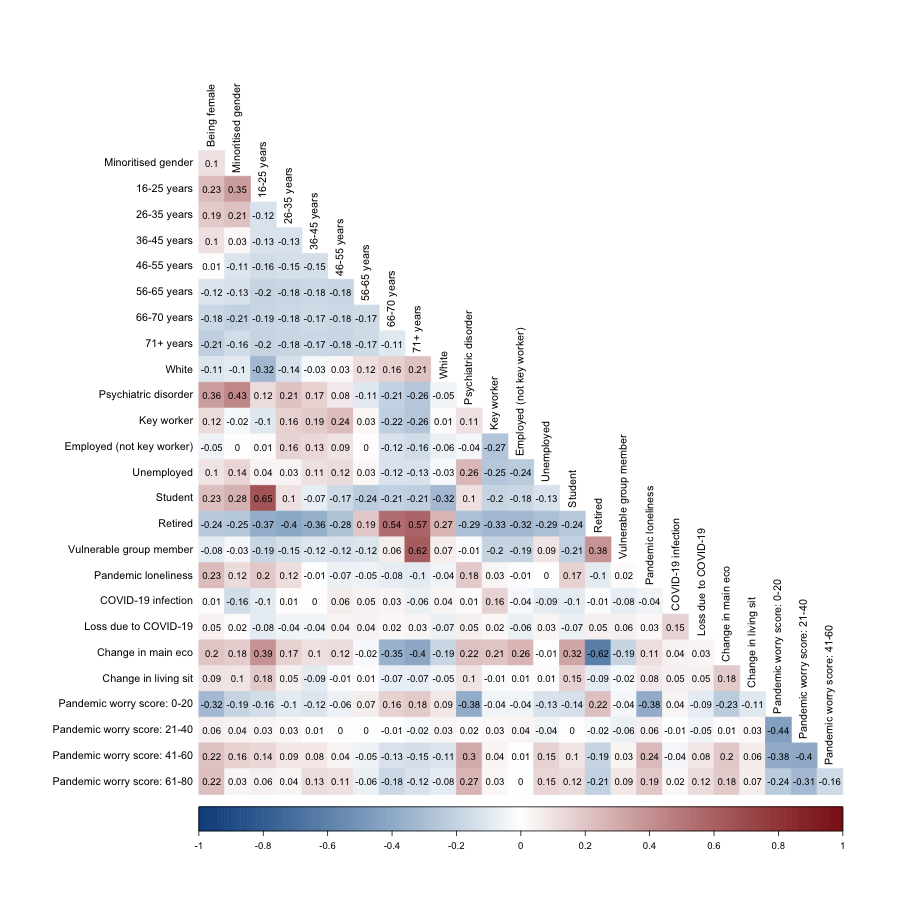


**SUPPLEMENTARY FIGURE 3** Correlation matrix for all exposures included in the regression model with the outcome low weight (*n* = 24,364). All correlations are tetrachoric correlations. Note. Minoritised gender = ‘Transgender’, ‘Non-binary’, and ‘Prefer to self-define’; Racially minoritised = ‘Arab’, ‘Asian’, ‘Black’, ‘Mixed race’, and ‘Other’.

**
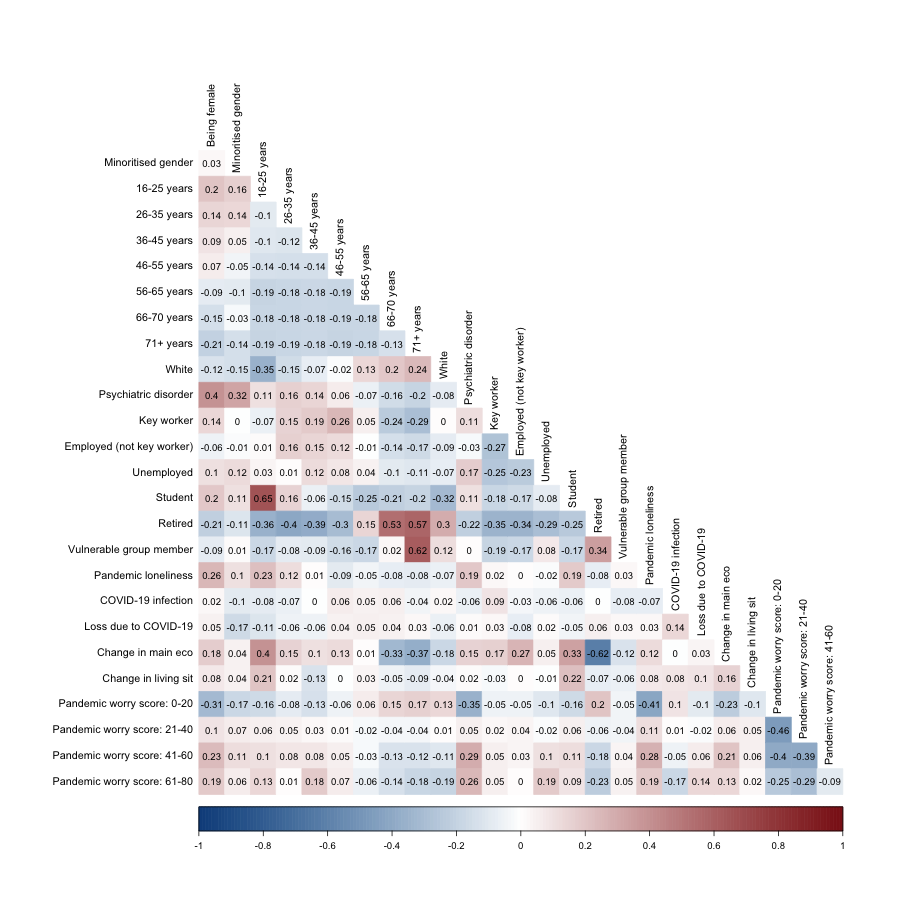
SUPPLEMENTARY FIGURE 4** Correlation matrix for all exposures included in the regression model with the outcome suicidal and/or self-harm ideation (*n* = 18,040). All correlations are tetrachoric correlations. Note. Minoritised gender = ‘Transgender’, ‘Non-binary’, and ‘Prefer to self-define’; Racially minoritised = ‘Arab’, ‘Asian’, ‘Black’, ‘Mixed race’, and ‘Other’.

**
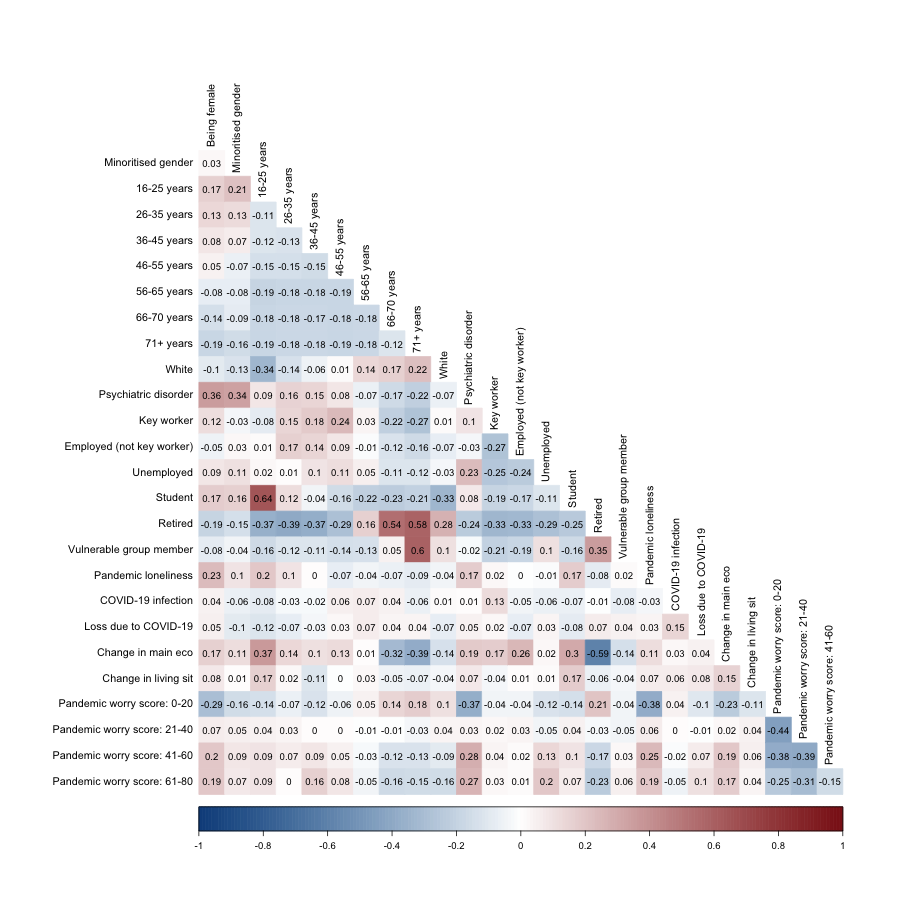
SUPPLEMENTARY FIGURE 5** Correlation matrix for all exposures included in the regression model with the outcome self-harm (*n* = 29,948). All correlations are tetrachoric correlations. Note. Minoritised gender = ‘Transgender’, ‘Non-binary’, and ‘Prefer to self-define’; Racially minoritised = ‘Arab’, ‘Asian’, ‘Black’, ‘Mixed race’, and ‘Other’.

**1.3 Assessing assumptions**

Below, we have displayed the histograms and Q-Q plots for BMI at registration within the whole sample and within each subsample defined by outcome, calculated to assess normality. BMI at registration violated the assumption of normality, thus we reported medians and interquartile range (IQR).


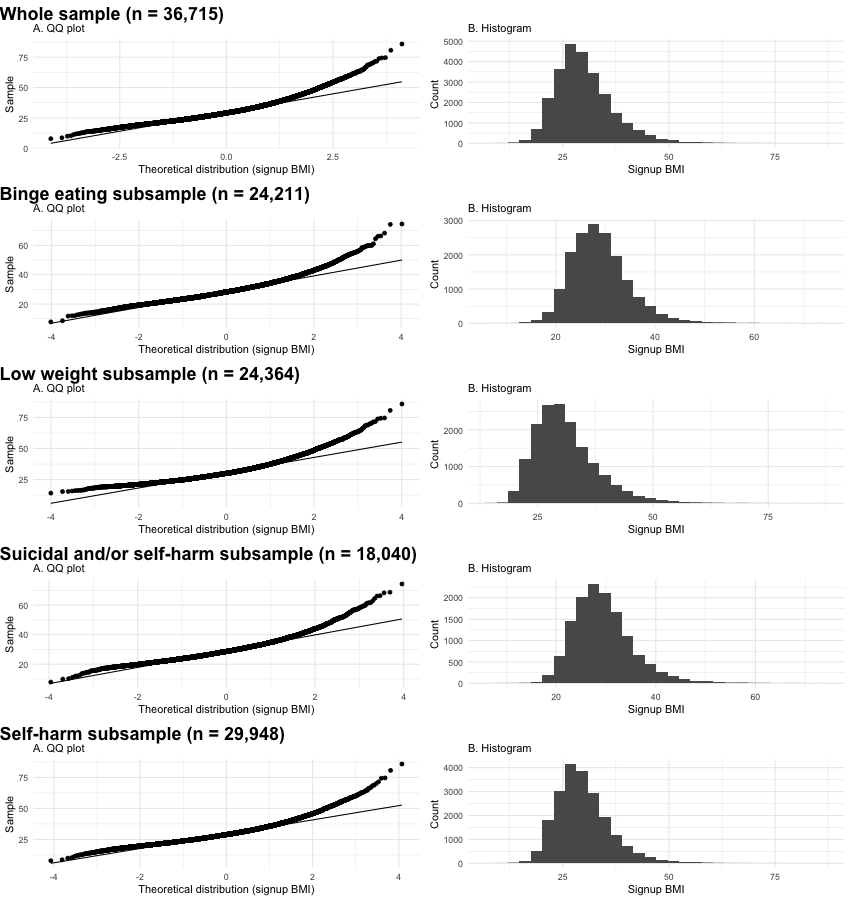


**SUPPLEMENTARY FIGURE 6** Histogram and Q-Q plots of BMI at registration in the whole sample (*n* = 36,715) and in each subsample defined by outcome: binge eating (*n* = 24,211), low weight (*n* = 24,364), suicidal and/or self-harm ideation (*n* = 18,040), and self-harm (*n* = 29,948).

**2 SUPPLEMENTARY RESULTS**

**2.1 *N* of new onset split by analyses**

**SUPPLEMENTARY TABLE 2** Number and percentage of participants with new onset in each of the subsamples defined by outcome: binge eating, low weight, suicidal and/or self-harm ideation, and self-harm in the main analysis and each sensitivity analysis. Participants are from National Institute for Health and Care Research (NIHR) BioResource sub-cohorts who joined the COVID-19 Psychiatry and Neurological Genetics (COPING) study, or the Repeated Assessment of Mental health in Pandemics (RAMP) Study. Participants reported new onset of each symptom in the COPING study or the RAMP Study.

|  | **N with new onset between April 2020 and July 2021 (%)** | | | |
| --- | --- | --- | --- | --- |
|  | **Binge eating** | **Low weight** | **Suicidal and/or self-harm ideation** | **Self-harm** |
| **Main analysis** | 5,080/24,211 (21.0%) | 2,636/24,364  (10.8%) | 4,246/18,040  (23.5%) | 1,039/29,948  (3.5%) |
| **Sensitivity analysis A** | 3,602/18,323 (19.7%) | 1,920/18,091 (10.6%) | 2,870/15,342 (18.7%) | 565/22,504  (2.5%) |
| **Sensitivity analysis B** | 4,688/22,016 (21.3%) | 2,430/22,747 (10.7%) | 3,870/15,849 (24.4%) | 972/26,759  (3.6%) |
| **Sensitivity analysis C** | 5,080/19,500 (26.1%) | 2,636/18,509 (14.3%) | 4,246/14,470 (29.3%) | 1,039/22,849 (4.5%) |
| **Sensitivity analysis D** | 4,619/23,901 (19.3%) | 2,355/24,222 (9.7%) | 2,809/17,433 (16.1%) | 906/29,867  (3.0%) |

*Note.* Sensitivity analysis A = analysis in which we excluded participants ascertained for having a psychiatric disorder, i.e., EDGI UK and GLAD survey participants; Sensitivity analysis B = analysis in which we excluded participants with inflammatory bowel disease; Sensitivity analysis C = analysis in which we specified that participants must have answered ‘No’ at least three times to be classified as having not experienced the relevant outcome during the monitoring period; Sensitivity analysis D = analysis in which we dropped all instances in which the exposure was first reported in the same phase or a phase after the first report of the outcome.

**2.2 *N* of exposure split by outcome**

**SUPPLEMENTARY TABLE 3** Number and percentage of participants in the binge eating subsample (*n* = 24,211) with and without each exposure included in the main analysis, split by those with and without new onset of the outcome.

|  | **N with new onset binge eating (%)** | **N with no new onset binge eating (%)** |
| --- | --- | --- |
| **Age (years)** |  |  |
| 46-55 (reference category) | 1165 (24.0%) | 3682 (76.0%) |
| 16-25 | 420 (24.0%) | 1332 (76.0%) |
| 26-35 | 586 (23.1%) | 1952 (76.9%) |
| 36-45 | 686 (24.2%) | 2150 (75.8%) |
| 56-65 | 1361 (20.6%) | 5263 (79.4%) |
| 66-70 | 470 (16.4%) | 2399 (83.6%) |
| 71+ | 392 (14.3%) | 2353 (85.7%) |
| **Sex** |  |  |
| Male (reference category) | 1279 (16.3%) | 6554 (83.7%) |
| Female | 3780 (23.2%) | 12524 (76.8%) |
| **Minoritised gender** |  |  |
| Yes | 79 (27.2%) | 212 (72.8%) |
| No (reference category) | 4994 (20.9%) | 18890 (79.1%) |
| **Racially minoritised** |  |  |
| Yes | 215 (28.1%) | 550 (71.9%) |
| No (reference category) | 4779 (20.8%) | 18153 (79.2%) |
| **Psychiatric disorder** |  |  |
| Yes | 3417 (26.4%) | 9529 (73.6%) |
| No (reference category) | 1600 (14.6%) | 9361 (85.4%) |
| **Employment** |  |  |
| In paid employment (reference category) | 1117 (18.8%) | 4817 (81.2%) |
| Key worker | 1973 (24.2%) | 6180 (75.8%) |
| Not in paid employment | 671 (29.0%) | 1643 (71.0%) |
| Retired | 1107 (16.0%) | 5833 (84.0%) |
| Student | 202 (24.5%) | 624 (75.5%) |
| **Vulnerable group member** |  |  |
| Yes (reference category) | 1369 (21.6%) | 4965 (78.4%) |
| No | 3646 (20.6%) | 14043 (79.4%) |
| **Pandemic loneliness** |  |  |
| Yes | 2241 (24.4%) | 6959 (75.6%) |
| No (reference category) | 2826 (18.9%) | 12142 (81.1%) |
| **Pandemic worry score** |  |  |
| 0-20 (reference category) | 898 (13.5%) | 5775 (86.5%) |
| 21-40 | 2855 (21.1%) | 10678 (78.9%) |
| 41-60 | 1213 (33.2%) | 2446 (66.8%) |
| 61-80 | 80 (42.5%) | 108 (57.5%) |
| **COVID-19 infection** |  |  |
| Yes | 102 (8.0%) | 1168 (92.0%) |
| No (reference category) | 4664 (20.6%) | 17939 (79.4%) |
| **Loss of loved one/relative due to COVID-19** |  |  |
| Yes | 353 (19.0%) | 1506 (81.0%) |
| No (reference category) | 4426 (20.1%) | 17605 (79.9%) |
| **Change in main economic activity** |  |  |
| Yes | 2057 (21.6%) | 7465 (78.4%) |
| No (reference category) | 2626 (18.5%) | 11575 (81.5%) |
| **Change in living situation** |  |  |
| Yes | 940 (17.5%) | 4419 (82.5%) |
| No (reference category) | 3689 (20.1%) | 14712 (79.9%) |

*Note.* Minoritised gender = ‘Transgender’, ‘Non-binary’, and ‘Prefer to self-define’; Racially minoritised = ‘Arab’, ‘Asian’, ‘Black’, ‘Mixed race’, and ‘Other’.

**SUPPLEMENTARY TABLE 4** Number and percentage of participants in the low weight subsample (*n* = 24,364) with and without each exposure included in the main analysis, split by those with and without new onset of the outcome.

|  | **N with new onset low weight (%)** | **N with no new onset low weight (%)** |
| --- | --- | --- |
| **Age (years)** |  |  |
| 46-55 (reference category) | 568 (11.4%) | 4412 (88.6%) |
| 16-25 | 279 (14.7%) | 1618 (85.3%) |
| 26-35 | 241 (8.1%) | 2718 (91.9%) |
| 36-45 | 305 (10.1%) | 2713 (89.9%) |
| 56-65 | 714 (11.3%) | 5616 (88.7%) |
| 66-70 | 254 (9.6%) | 2407 (90.4%) |
| 71+ | 275 (10.9%) | 2244 (89.1%) |
| **Sex** |  |  |
| Female (reference category) | 1763 (10.6%) | 14930 (89.4%) |
| Male | 862 (11.4%) | 6716 (88.6%) |
| **Minoritised gender** |  |  |
| Yes | 47 (13.4%) | 304 (86.6%) |
| No (reference category) | 2585 (10.8%) | 21389 (89.2%) |
| **Racially minoritised** |  |  |
| Yes | 118 (14.6%) | 689 (85.4%) |
| No (reference category) | 2467 (10.7%) | 20588 (89.3%) |
| **Psychiatric disorder** |  |  |
| Yes | 1736 (12.5%) | 12177 (87.5%) |
| No (reference category) | 865 (8.5%) | 9299 (91.5%) |
| **Employment** |  |  |
| In paid employment (reference category) | 516 (8.8%) | 5332 (91.2%) |
| Key worker | 934 (10.9%) | 7626 (89.1%) |
| Not in paid employment | 391 (15.4%) | 2151 (84.6%) |
| Retired | 646 (9.9%) | 5849 (90.1%) |
| Student | 143 (16.3%) | 733 (83.7%) |
| **Vulnerable group member** |  |  |
| Yes (reference category) | 869 (14.3%) | 5198 (85.7%) |
| No | 1740 (9.6%) | 16365 (90.4%) |
| **Pandemic loneliness** |  |  |
| Yes | 1149 (11.9%) | 8523 (88.1%) |
| No (reference category) | 1478 (10.1%) | 13168 (89.9%) |
| **Pandemic worry score** |  |  |
| 0-20 (reference category) | 491 (7.9%) | 5745 (92.1%) |
| 21-40 | 1402 (10.3%) | 12180 (89.7%) |
| 41-60 | 664 (16.0%) | 3485 (84.0%) |
| 61-80 | 57 (23.4%) | 187 (76.6%) |
| **COVID-19 infection** |  |  |
| Yes | 78 (5.5%) | 1344 (94.5%) |
| No (reference category) | 2417 (10.6%) | 20314 (89.4%) |
| **Loss of loved one/relative due to COVID-19** |  |  |
| Yes | 212 (10.5%) | 1812 (89.5%) |
| No (reference category) | 2270 (10.3%) | 19848 (89.7%) |
| **Change in main economic activity** |  |  |
| Yes | 1047 (10.4%) | 8978 (89.6%) |
| No (reference category) | 1384 (9.9%) | 12627 (90.1%) |
| **Change in living situation** |  |  |
| Yes | 549 (9.7%) | 5119 (90.3%) |
| No (reference category) | 1844 (10.0%) | 16609 (90.0%) |

Note. Minoritised gender = ‘Transgender’, ‘Non-binary’, and ‘Prefer to self-define’; Racially minoritised = ‘Arab’, ‘Asian’, ‘Black’, ‘Mixed race’, and ‘Other’.

**SUPPLEMENTARY TABLE 5** Number and percentage of participants in the suicidal and/or self-harm ideation subsample (*n* = 18,040) with and without each exposure included in the main analysis, split by those with and without new onset of the outcome.

|  | **N with new onset suicidal and/or self-harm ideation (%)** | **N with no new onset suicidal and/or self-harm ideation (%)** |
| --- | --- | --- |
| **Age (years)** |  |  |
| 46-55 (reference category) | 877 (24.3%) | 2726 (75.7%) |
| 16-25 | 366 (38.0%) | 598 (62.0%) |
| 26-35 | 547 (29.9%) | 1282 (70.1%) |
| 36-45 | 524 (25.5%) | 1529 (74.5%) |
| 56-65 | 1134 (22.6%) | 3881 (77.4%) |
| 66-70 | 400 (17.2%) | 1926 (82.8%) |
| 71+ | 398 (17.7%) | 1852 (82.3%) |
| **Sex** |  |  |
| Female (reference category) | 3195 (27.5%) | 8431 (72.5%) |
| Male | 1039 (16.3%) | 5350 (83.7%) |
| **Minoritised gender** |  |  |
| Yes | 53 (55.8%) | 42 (44.2%) |
| No (reference category) | 4185 (23.3%) | 13746 (76.7%) |
| **Racially minoritised** |  |  |
| Yes | 191 (33.8%) | 375 (66.2%) |
| No (reference category) | 3985 (23.4%) | 13017 (76.6%) |
| **Psychiatric disorder** |  |  |
| Yes | 2915 (40.1%) | 4359 (59.9%) |
| No (reference category) | 1233 (11.8%) | 9197 (88.2%) |
| **Employment** |  |  |
| In paid employment (reference category) | 1030 (23.2%) | 3417 (76.8%) |
| Key worker | 1573 (24.9%) | 4749 (75.1%) |
| Not in paid employment | 430 (34.8%) | 805 (65.2%) |
| Retired | 1047 (18.7%) | 4537 (81.3%) |
| Student | 156 (36.9%) | 267 (63.1%) |
| **Vulnerable group member** |  |  |
| Yes (reference category) | 1136 (23.1%) | 3787 (76.9%) |
| No | 3058 (23.6%) | 9900 (76.4%) |
| **Pandemic loneliness** |  |  |
| Yes | 1913 (30.6%) | 4341 (69.4%) |
| No (reference category) | 2196 (19.5%) | 9066 (80.5%) |
| **Pandemic worry score** |  |  |
| 0-20 (reference category) | 786 (13.7%) | 4969 (86.3%) |
| 21-40 | 2382 (24.9%) | 7176 (75.1%) |
| 41-60 | 864 (42.1%) | 1187 (57.9%) |
| 61-80 | 64 (53.3%) | 56 (46.7%) |
| **COVID-19 infection** |  |  |
| Yes | 46 (5.9%) | 733 (94.1%) |
| No (reference category) | 3753 (23.2%) | 12434 (76.8%) |
| **Loss of loved one/relative due to COVID-19** |  |  |
| Yes | 206 (17.1%) | 996 (82.9%) |
| No (reference category) | 3611 (22.9%) | 12169 (77.1%) |
| **Change in main economic activity** |  |  |
| Yes | 1563 (25.6%) | 4542 (74.4%) |
| No (reference category) | 2213 (20.2%) | 8726 (79.8%) |
| **Change in living situation** |  |  |
| Yes | 607 (19.2%) | 2553 (80.8%) |
| No (reference category) | 3138 (21.8%) | 11240 (78.2%) |

Note. Minoritised gender = ‘Transgender’, ‘Non-binary’, and ‘Prefer to self-define’; Racially minoritised = ‘Arab’, ‘Asian’, ‘Black’, ‘Mixed race’, and ‘Other’.

**SUPPLEMENTARY TABLE 6** Number and percentage of participants in the self-harm subsample (*n* = 29,948) with and without each exposure included in the main analysis, split by those with and without new onset of the outcome.

|  | **N with new onset self-harm (%)** | **N with no new onset self-harm (%)** |
| --- | --- | --- |
| **Age (years)** |  |  |
| 46-55 (reference category) | 218 (3.5%) | 6090 (96.5%) |
| 16-25 | 156 (8.6%) | 1650 (91.4%) |
| 26-35 | 211 (6.5%) | 3059 (93.5%) |
| 36-45 | 157 (4.2%) | 3612 (95.8%) |
| 56-65 | 188 (2.3%) | 7955 (97.7%) |
| 66-70 | 60 (1.8%) | 3349 (98.2%) |
| 71+ | 49 (1.5%) | 3192 (98.5%) |
| **Sex** |  |  |
| Female (reference category) | 778 (3.9%) | 19329 (96.1%) |
| Male | 255 (2.6%) | 9525 (97.4%) |
| **Minoritised gender** |  |  |
| Yes | 23 (9.2%) | 226 (90.8%) |
| No (reference category) | 1012 (3.4%) | 28652 (96.6%) |
| **Racially minoritised** |  |  |
| Yes | 49 (4.8%) | 978 (95.2%) |
| No (reference category) | 975 (3.5%) | 27289 (96.5%) |
| **Psychiatric disorder** |  |  |
| Yes | 805 (5.0%) | 15414 (95.0%) |
| No (reference category) | 214 (1.6%) | 12971 (98.4%) |
| **Employment** |  |  |
| In paid employment (reference category) | 253 (3.5%) | 7089 (96.5%) |
| Key worker | 401 (3.7%) | 10383 (96.3%) |
| Not in paid employment | 162 (6.0%) | 2540 (94.0%) |
| Retired | 148 (1.8%) | 8088 (98.2%) |
| Student | 72 (8.8%) | 751 (91.2%) |
| **Vulnerable group member** |  |  |
| Yes (reference category) | 261 (3.2%) | 7789 (96.8%) |
| No | 768 (3.5%) | 20865 (96.5%) |
| **Pandemic loneliness** |  |  |
| Yes | 501 (4.6%) | 10474 (95.4%) |
| No (reference category) | 509 (2.8%) | 17430 (97.2%) |
| **Pandemic worry score** |  |  |
| 0-20 (reference category) | 169 (2.1%) | 7744 (97.9%) |
| 21-40 | 549 (3.4%) | 15656 (96.6%) |
| 41-60 | 262 (5.8%) | 4228 (94.2%) |
| 61-80 | 26 (9.1%) | 259 (90.9%) |
| **COVID-19 infection** |  |  |
| Yes | 28 (1.8%) | 1544 (98.2%) |
| No (reference category) | 916 (3.4%) | 25721 (96.6%) |
| **Loss of loved one/relative due to COVID-19** |  |  |
| Yes | 83 (3.6%) | 2227 (96.4%) |
| No (reference category) | 868 (3.4%) | 25046 (96.6%) |
| **Change in main economic activity** |  |  |
| Yes | 492 (4.2%) | 11172 (95.8%) |
| No (reference category) | 478 (2.8%) | 16797 (97.2%) |
| **Change in living situation** |  |  |
| Yes | 229 (3.6%) | 6088 (96.4%) |
| No (reference category) | 724 (3.1%) | 22818 (96.9%) |

Note. Minoritised gender = ‘Transgender’, ‘Non-binary’, and ‘Prefer to self-define’; Racially minoritised = ‘Arab’, ‘Asian’, ‘Black’, ‘Mixed race’, and ‘Other’.

**2.3 Sensitivity analysis results**

**Excluding GLAD Study and EDGI UK survey participants.** We first compared our logistic regression results from our main analysis to the results from the sensitivity analyses in which we excluded participants not ascertained for having a psychiatric disorder (binge eating *n* = 18,323; low weight *n* = 18,091; suicidal and/or self-harm ideation *n* = 15,342; self-harm *n* = 22,504; Supplementary Figures 7-10). The odds ratios of both analyses were highly correlated across all models (*r* = 0.92-0.98), indicating that our analyses were not sensitive to ascertainment bias in terms of psychiatric disorders.

**Excluding participants with IBD.** Next, we compared our main logistic regression results to the results from the sensitivity analyses (Supplementary Figures 11-14) in which we excluded participants from the inflammatory bowel disease cohort (*n* = 2,714) and who self-reported a diagnosis of inflammatory bowel disease (*n* = 3,319). This resulted in the following sample sizes: binge eating *n* = 22,016; low weight *n* = 22,747; suicidal and/or self-harm ideation *n* = 15,849; self-harm *n* = 26,759. The odds ratios of both analyses were highly correlated across all models (all *r*s = 0.99), indicating that our analyses were not sensitive to ascertainment bias in terms of inflammatory bowel disease.

**Missing data.** Next, we compared our main analysis results to the results from the sensitivity analyses in which we specified that participants must have answered ‘*No*’ at least three times to be considered to not have experienced the outcome during the monitoring period (binge eating *n* = 19,500; low weight *n* = 18,509; suicidal and/or self-harm ideation *n* = 14,470; self-harm *n* = 22,849; Supplementary Figures 15-18). The odds ratios of both analyses were highly correlated across all models (*r* = 0.93-0.97), indicating that our analyses were not sensitive to missing data.

**Exposure before outcome.** Finally, we compared our main analysis results to our results from another sensitivity analysis whereby, where appropriate, we only included instances in which the outcome was first reported in a phase after the exposure was first reported (binge eating *n* = 23,901; low weight *n* = 24,222; suicidal and/or self-harm ideation *n* = 17,433; self-harm *n* = 29,867; Supplementary Figures 19-22). The odds ratios of both analyses were highly correlated across all models (*r* = 0.97-0.99), indicating that our analyses were robust to attempts to further limit the possibility of reverse causality.

**
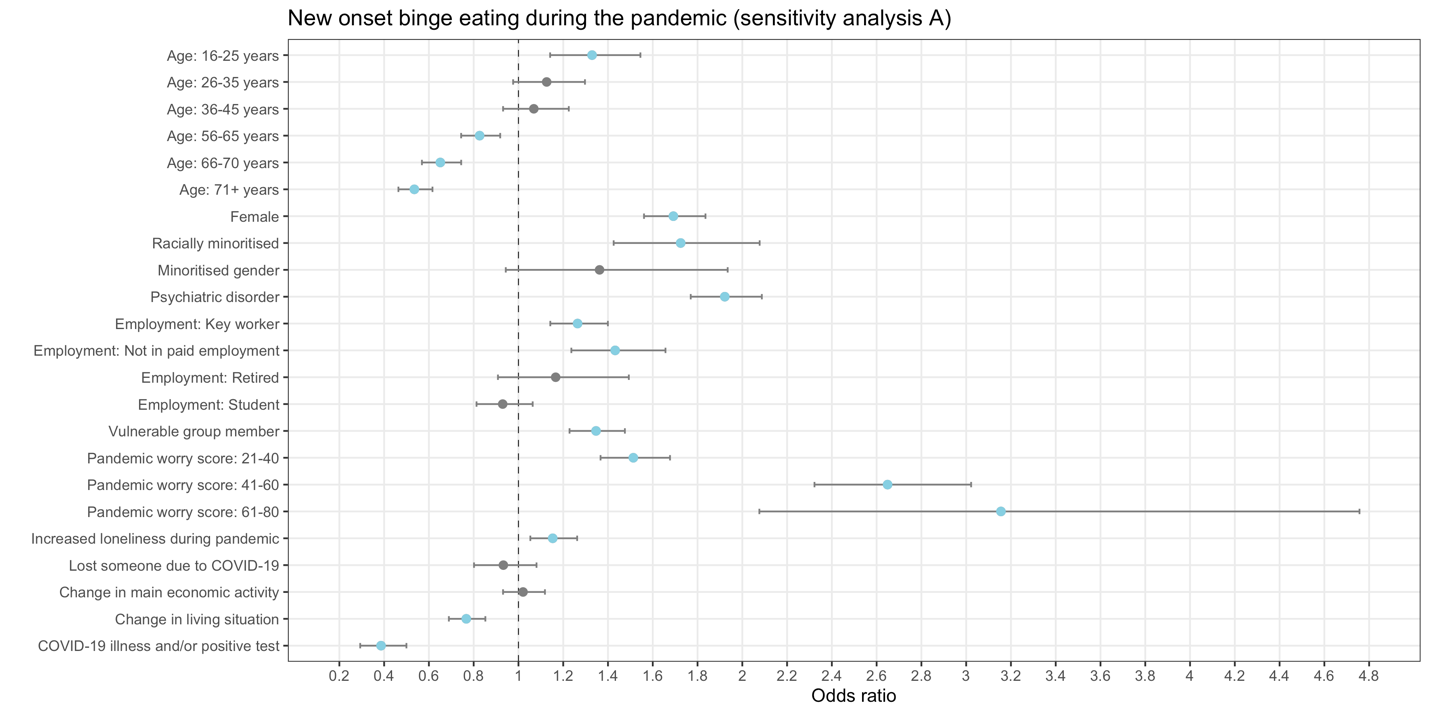
SUPPLEMENTARY FIGURE 7** Association between demographic and COVID-related variables and new onset of binge eating during the pandemic. Results are derived from multiple logistic regression models in a sensitivity analysis in which we excluded participants ascertained for having a psychiatric disorder (*n* = 18,323). Points in blue are significant (*p* < 0.0038). We found a high correlation between the odds ratios from this analysis with the odds ratios from the main analysis (*r* = 0.98). *Note*. Minoritised gender = ‘Transgender’, ‘Non-binary’, and ‘Prefer to self-define’; Racially minoritised = ‘Arab’, ‘Asian’, ‘Black’, ‘Mixed race’, and ‘Other’. Reference category for age is 46-55 years, for female is being male, for racially minoritised is being white, for minoritised gender is not being minoritised gender, for psychiatric disorder is not having a psychiatric disorder, for employment is being in paid employment, for vulnerable group member is not being a vulnerable group member, for pandemic worry score is a score of 0-20, for losing someone due to COVID-19 is not losing someone due to COVID-19, for change in main economic activity is not experiencing a change in main economic activity, and for change in living situation is not experiencing a change in living situation.


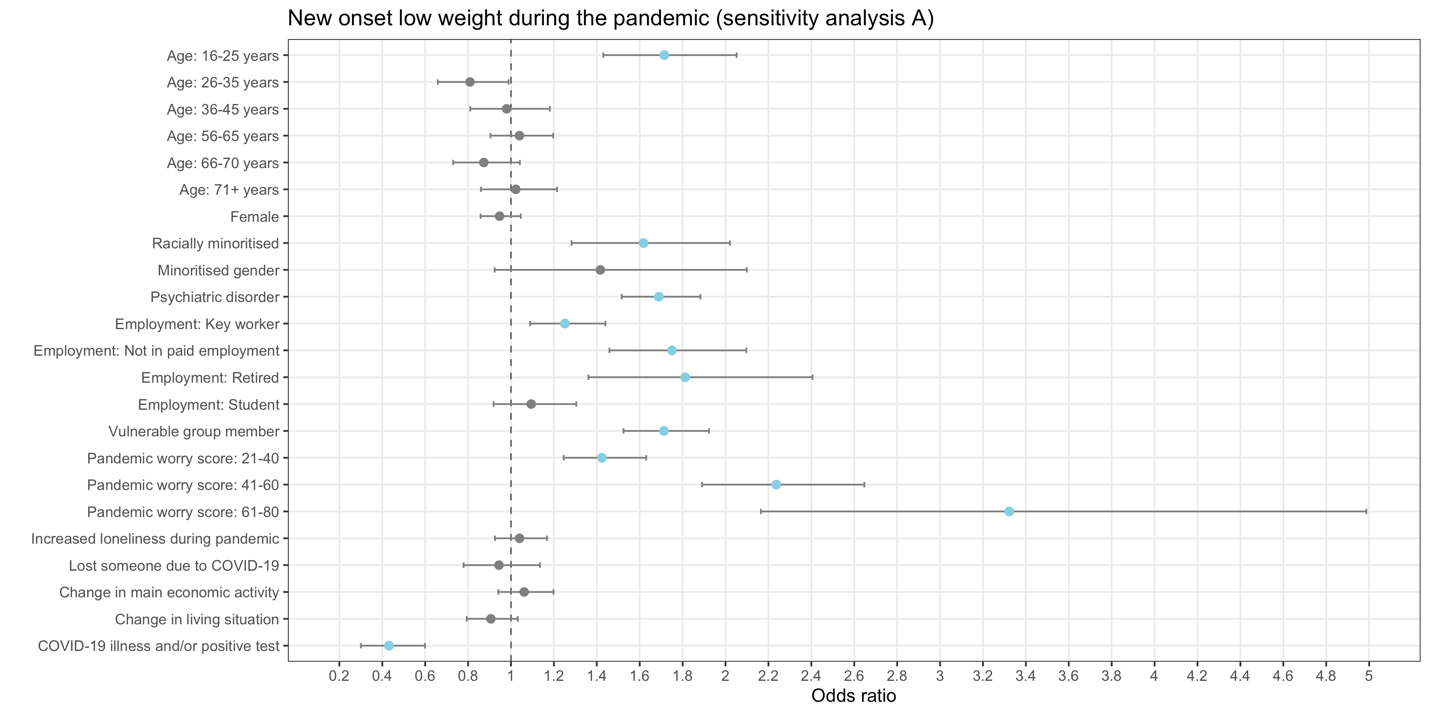


**SUPPLEMENTARY FIGURE 8** Association between demographic and COVID-related variables and new onset of low weight during the pandemic. Results are derived from multiple logistic regression models in a sensitivity analysis in which we excluded participants ascertained for having a psychiatric disorder (*n* = 18,091). Points in blue are significant (*p* < 0.0038). We found a high correlation between the odds ratios from this analysis with the odds ratios from the main analysis (*r* = 0.92). *Note*. Minoritised gender = ‘Transgender’, ‘Non-binary’, and ‘Prefer to self-define’; Racially minoritised = ‘Arab’, ‘Asian’, ‘Black’, ‘Mixed race’, and ‘Other’. Reference category for age is 46-55 years, for female is being male, for racially minoritised is being white, for minoritised gender is not being minoritised gender, for psychiatric disorder is not having a psychiatric disorder, for employment is being in paid employment, for vulnerable group member is not being a vulnerable group member, for pandemic worry score is a score of 0-20, for losing someone due to COVID-19 is not losing someone due to COVID-19, for change in main economic activity is not experiencing a change in main economic activity, and for change in living situation is not experiencing a change in living situation.

**
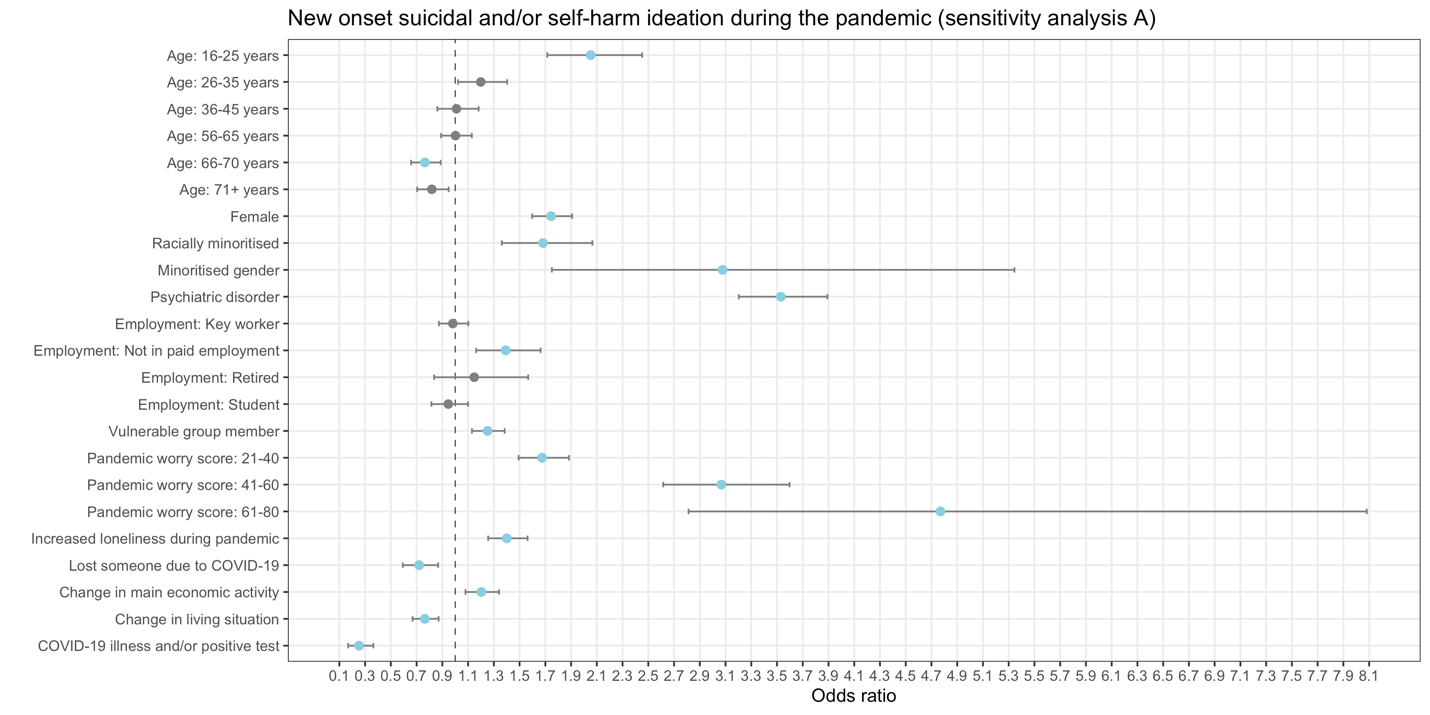
**

**SUPPLEMENTARY FIGURE 9** Association between demographic and COVID-related variables and new onset of suicidal and/or self-harm ideation during the pandemic. Results are derived from multiple logistic regression models in a sensitivity analysis in which we excluded participants ascertained for having a psychiatric disorder (*n* = 15,342). Points in blue are significant (*p* < 0.0038). We found a high correlation between the odds ratios from this analysis with the odds ratios from the main analysis (*r* = 0.95). *Note.* Minoritised gender = ‘Transgender’, ‘Non-binary’, and ‘Prefer to self-define’; Racially minoritised = ‘Arab’, ‘Asian’, ‘Black’, ‘Mixed race’, and ‘Other’. Reference category for age is 46-55 years, for female is being male, for racially minoritised is being white, for minoritised gender is not being minoritised gender, for psychiatric disorder is not having a psychiatric disorder, for employment is being in paid employment, for vulnerable group member is not being a vulnerable group member, for pandemic worry score is a score of 0-20, for losing someone due to COVID-19 is not losing someone due to COVID-19, for change in main economic activity is not experiencing a change in main economic activity, and for change in living situation is not experiencing a change in living situation.

**
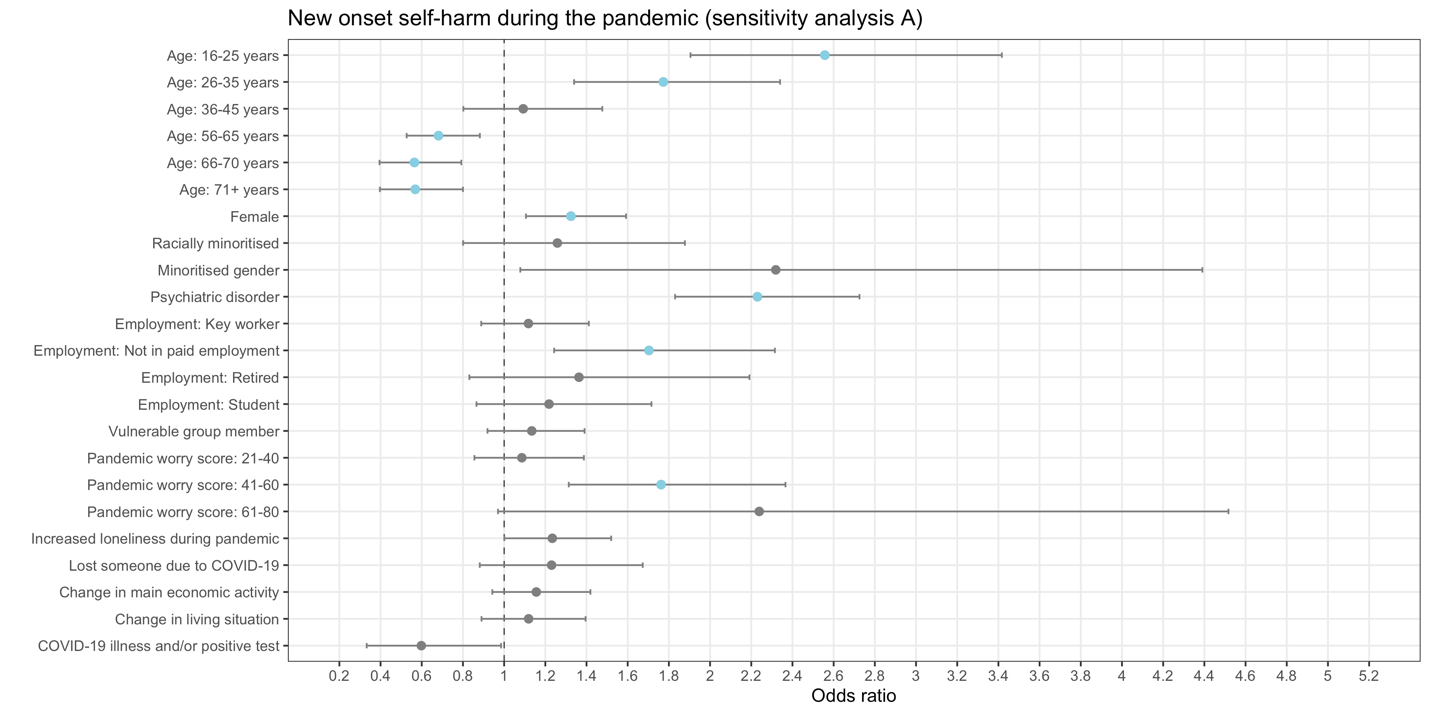
SUPPLEMENTARY FIGURE 10** Association between demographic and COVID-related variables and new onset of self-harm during the pandemic. Results are derived from multiple logistic regression models in a sensitivity analysis in which we excluded participants ascertained for having a psychiatric disorder (*n* = 22,504). Points in blue are significant (*p* < 0.0038). We found a high correlation between the odds ratios from this analysis with the odds ratios from the main analysis (*r* = 0.96). *Note*. Minoritised gender = ‘Transgender’, ‘Non-binary’, and ‘Prefer to self-define’; Racially minoritised = ‘Arab’, ‘Asian’, ‘Black’, ‘Mixed race’, and ‘Other’. Reference category for age is 46-55 years, for female is being male, for racially minoritised is being white, for minoritised gender is not being minoritised gender, for psychiatric disorder is not having a psychiatric disorder, for employment is being in paid employment, for vulnerable group member is not being a vulnerable group member, for pandemic worry score is a score of 0-20, for losing someone due to COVID-19 is not losing someone due to COVID-19, for change in main economic activity is not experiencing a change in main economic activity, and for change in living situation is not experiencing a change in living situation.

**
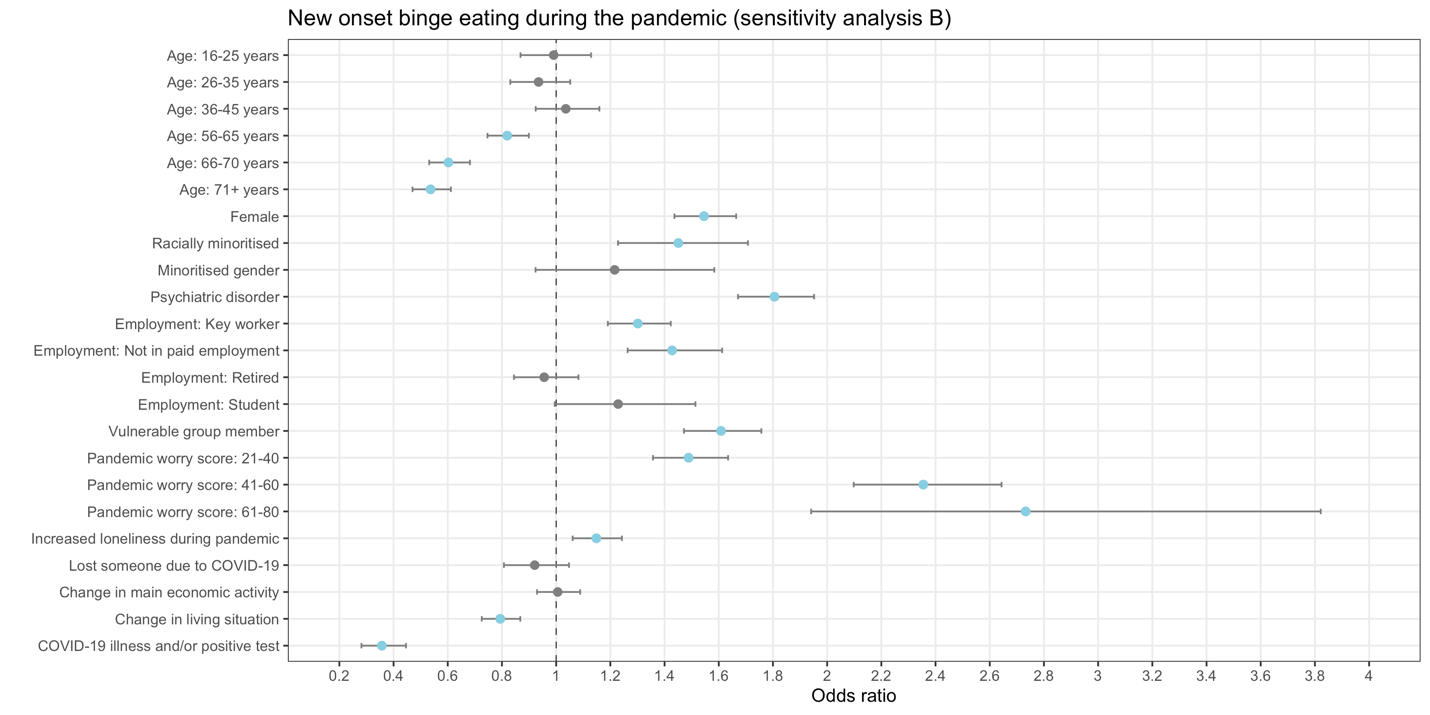
SUPPLEMENTARY FIGURE 11** Association between demographic and COVID-related variables and new onset of binge eating during the pandemic. Results are derived from multiple logistic regression models in a sensitivity analysis in which we excluded individuals with inflammatory bowel disease (*n* = 22,016). Points in blue are significant (*p* < 0.0038). We found a high correlation between the odds ratios from this analysis with the odds ratios from the main analysis (*r* = 0.99). *Note.* Minoritised gender = ‘Transgender’, ‘Non-binary’, and ‘Prefer to self-define’; Racially minoritised = ‘Arab’, ‘Asian’, ‘Black’, ‘Mixed race’, and ‘Other’. Reference category for age is 46-55 years, for female is being male, for racially minoritised is being white, for minoritised gender is not being minoritised gender, for psychiatric disorder is not having a psychiatric disorder, for employment is being in paid employment, for vulnerable group member is not being a vulnerable group member, for pandemic worry score is a score of 0-20, for losing someone due to COVID-19 is not losing someone due to COVID-19, for change in main economic activity is not experiencing a change in main economic activity, and for change in living situation is not experiencing a change in living situation.

**
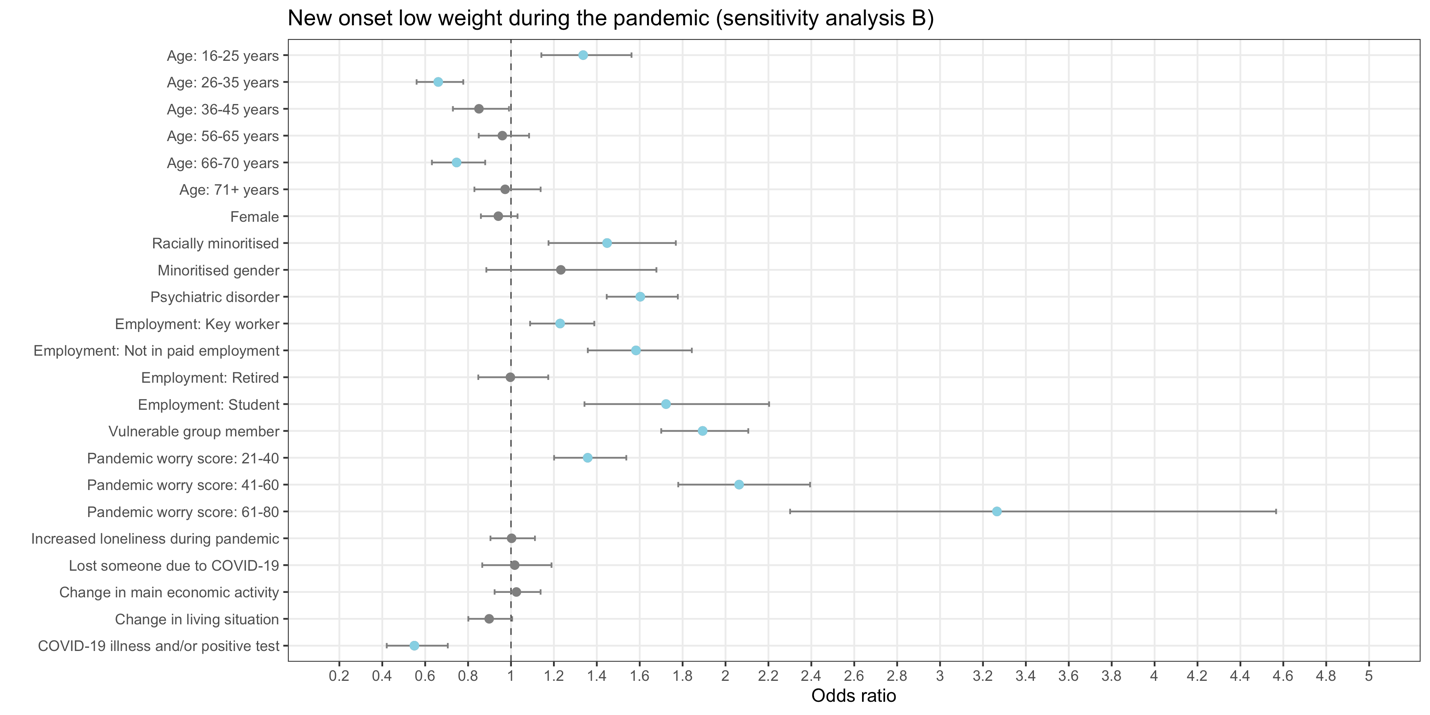
SUPPLEMENTARY FIGURE 12** Association between demographic and COVID-related variables and new onset of low weight during the pandemic. Results are derived from multiple logistic regression models in a sensitivity analysis in which we excluded individuals with inflammatory bowel disease (*n* = 22,747). Points in blue are significant (*p* < 0.0038). We found a high correlation between the odds ratios from this analysis with the odds ratios from the main analysis (*r* = 0.99). *Note*. Minoritised gender = ‘Transgender’, ‘Non-binary’, and ‘Prefer to self-define’; Racially minoritised = ‘Arab’, ‘Asian’, ‘Black’, ‘Mixed race’, and ‘Other’. Reference category for age is 46-55 years, for female is being male, for racially minoritised is being white, for minoritised gender is not being minoritised gender, for psychiatric disorder is not having a psychiatric disorder, for employment is being in paid employment, for vulnerable group member is not being a vulnerable group member, for pandemic worry score is a score of 0-20, for losing someone due to COVID-19 is not losing someone due to COVID-19, for change in main economic activity is not experiencing a change in main economic activity, and for change in living situation is not experiencing a change in living situation.


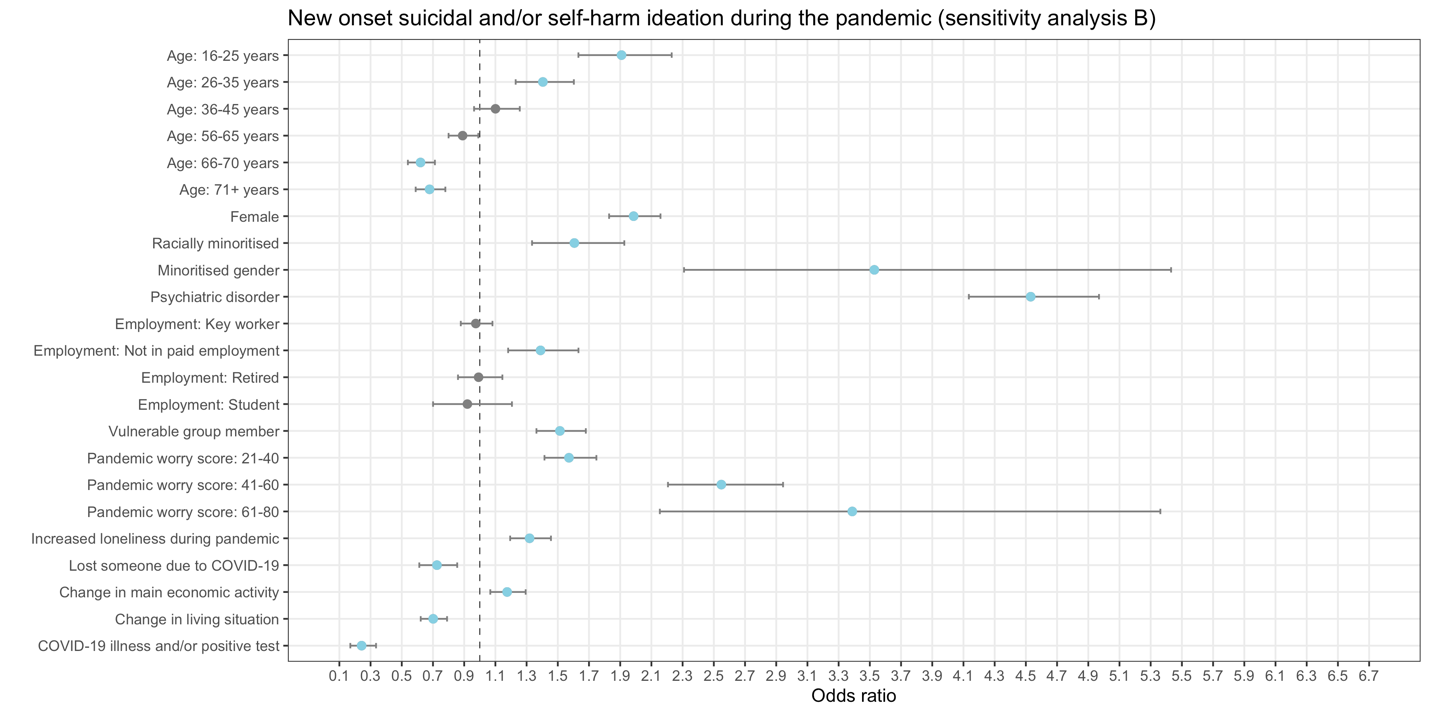


**SUPPLEMENTARY FIGURE 13** Association between demographic and COVID-related variables and new onset of suicidal and/or self-harm ideation during the pandemic. Results are derived from multiple logistic regression models in a sensitivity analysis in which we excluded individuals with inflammatory bowel disease (*n* = 15,849). Points in blue are significant (*p* < 0.0038). We found a high correlation between the odds ratios from this analysis with the odds ratios from the main analysis (*r* = 0.99). *Note*. Minoritised gender = ‘Transgender’, ‘Non-binary’, and ‘Prefer to self-define’; Racially minoritised = ‘Arab’, ‘Asian’, ‘Black’, ‘Mixed race’, and ‘Other’. Reference category for age is 46-55 years, for female is being male, for racially minoritised is being white, for minoritised gender is not being minoritised gender, for psychiatric disorder is not having a psychiatric disorder, for employment is being in paid employment, for vulnerable group member is not being a vulnerable group member, for pandemic worry score is a score of 0-20, for losing someone due to COVID-19 is not losing someone due to COVID-19, for change in main economic activity is not experiencing a change in main economic activity, and for change in living situation is not experiencing a change in living situation.

**
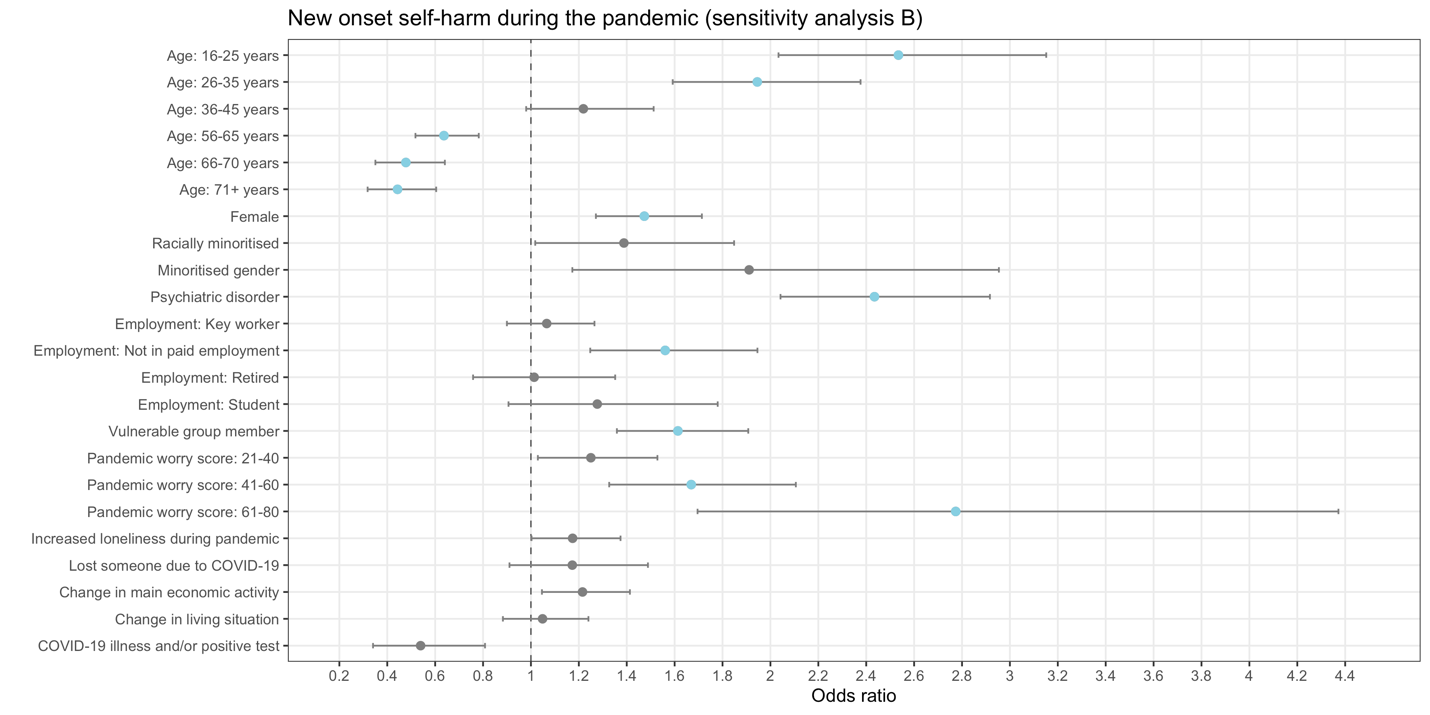
SUPPLEMENTARY FIGURE 14** Association between demographic and COVID-related variables and new onset of self-harm during the pandemic. Results are derived from multiple logistic regression models in a sensitivity analysis in which we excluded individuals with inflammatory bowel disease (*n* = 26,759). Points in blue are significant (*p* < 0.0038). We found a high correlation between the odds ratios from this analysis with the odds ratios from the main analysis (*r* = 0.99). *Note*. Minoritised gender = ‘Transgender’, ‘Non-binary’, and ‘Prefer to self-define’; Racially minoritised = ‘Arab’, ‘Asian’, ‘Black’, ‘Mixed race’, and ‘Other’. Reference category for age is 46-55 years, for female is being male, for racially minoritised is being white, for minoritised gender is not being minoritised gender, for psychiatric disorder is not having a psychiatric disorder, for employment is being in paid employment, for vulnerable group member is not being a vulnerable group member, for pandemic worry score is a score of 0-20, for losing someone due to COVID-19 is not losing someone due to COVID-19, for change in main economic activity is not experiencing a change in main economic activity, and for change in living situation is not experiencing a change in living situation.

**
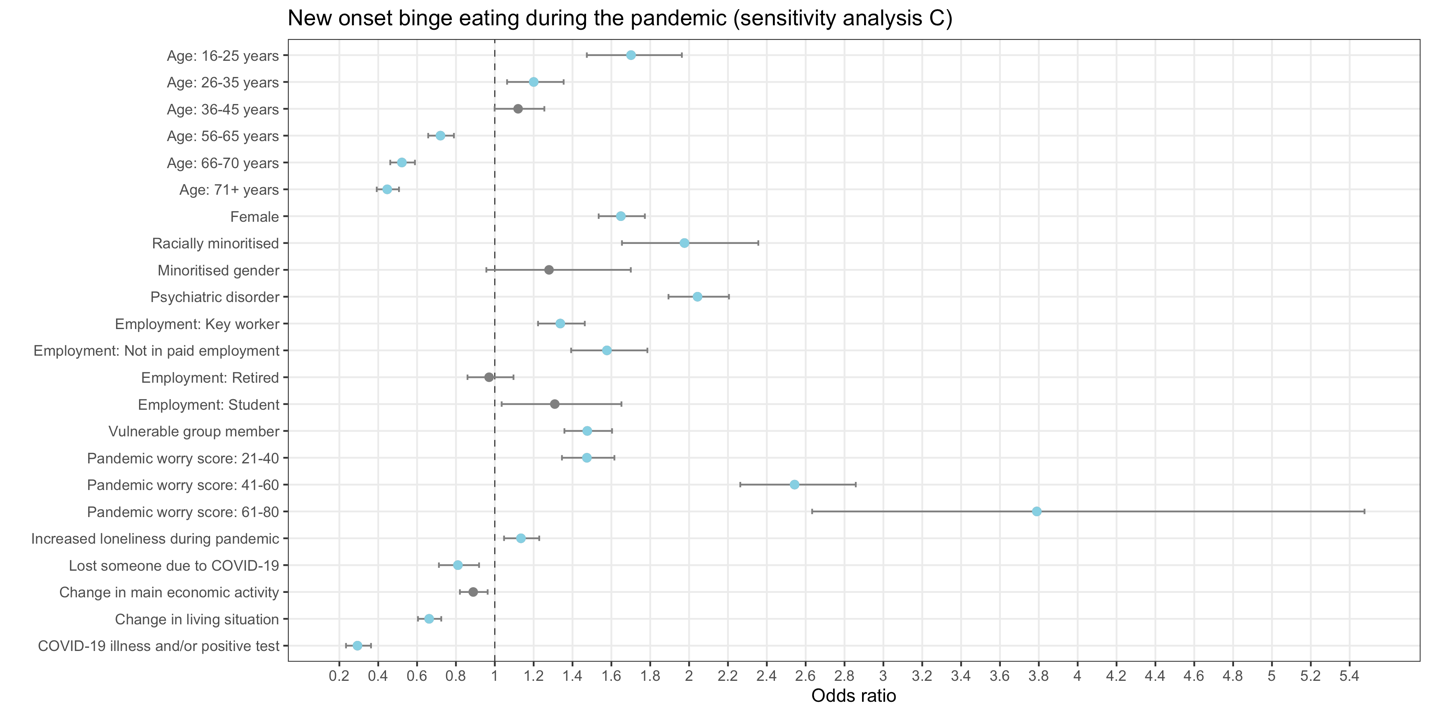
SUPPLEMENTARY FIGURE 15** Association between demographic and COVID-related variables and new onset of binge eating during the pandemic. Results are derived from multiple logistic regression models in a sensitivity analysis in which participants must have answered ‘No’ at least three times to be considered to not have experienced the outcome during the monitoring period (*n* = 19,500). Points in blue are significant (*p* < 0.0038). We found a high correlation between the odds ratios from this analysis with the odds ratios from the main analysis (*r* = 0.97). *Note*. Minoritised gender = ‘Transgender’, ‘Non-binary’, and ‘Prefer to self-define’; Racially minoritised = ‘Arab’, ‘Asian’, ‘Black’, ‘Mixed race’, and ‘Other’. Reference category for age is 46-55 years, for female is being male, for racially minoritised is being white, for minoritised gender is not being minoritised gender, for psychiatric disorder is not having a psychiatric disorder, for employment is being in paid employment, for vulnerable group member is not being a vulnerable group member, for pandemic worry score is a score of 0-20, for losing someone due to COVID-19 is not losing someone due to COVID-19, for change in main economic activity is not experiencing a change in main economic activity, and for change in living situation is not experiencing a change in living situation.

**
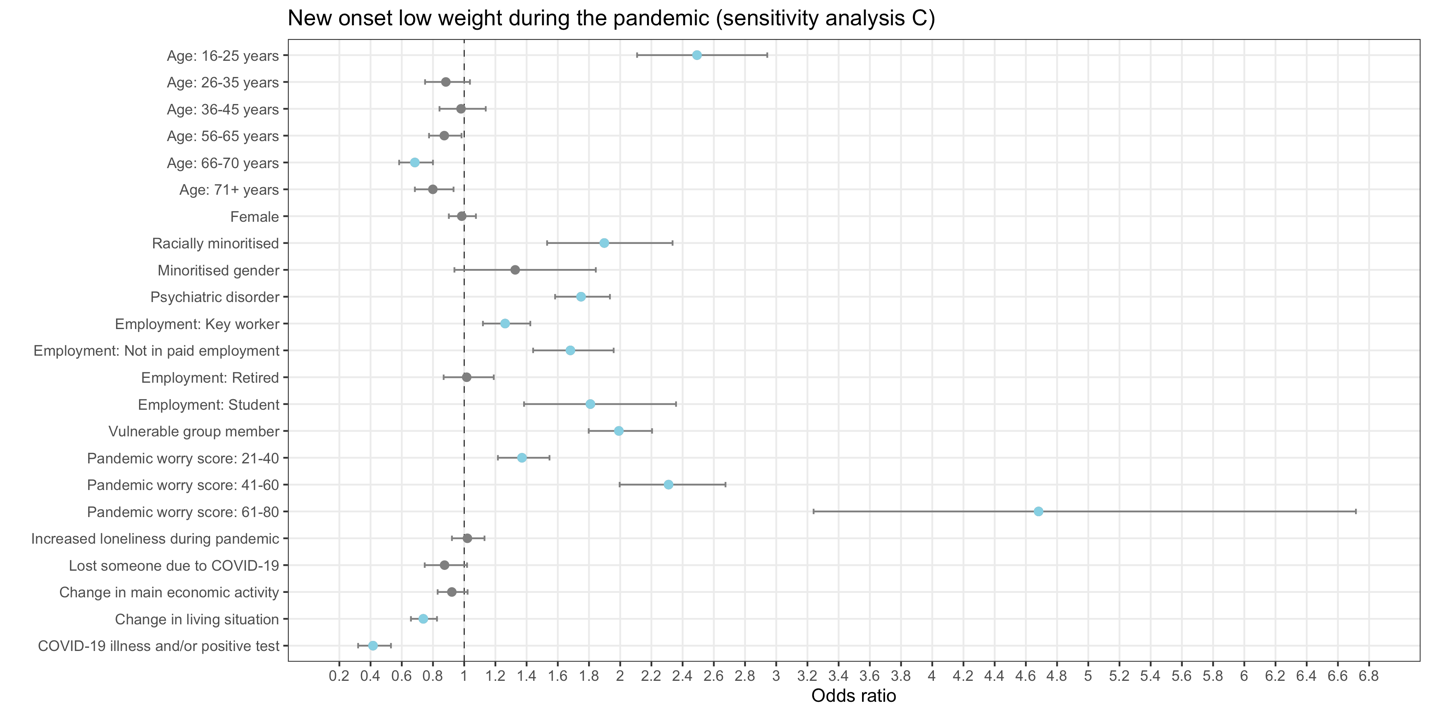
SUPPLEMENTARY FIGURE 16** Association between demographic and COVID-related variables and new onset of low weight during the pandemic. Results are derived from multiple logistic regression models in a sensitivity analysis in which participants must have answered ‘No’ at least three times to be considered to not have experienced the outcome during the monitoring period (*n* = 18,509). Points in blue are significant (*p* < 0.0038). We found a high correlation between the odds ratios from this analysis with the odds ratios from the main analysis (*r* = 0.95). *Note*. Minoritised gender = ‘Transgender’, ‘Non-binary’, and ‘Prefer to self-define’; Racially minoritised = ‘Arab’, ‘Asian’, ‘Black’, ‘Mixed race’, and ‘Other’. Reference category for age is 46-55 years, for female is being male, for racially minoritised is being white, for minoritised gender is not being minoritised gender, for psychiatric disorder is not having a psychiatric disorder, for employment is being in paid employment, for vulnerable group member is not being a vulnerable group member, for pandemic worry score is a score of 0-20, for losing someone due to COVID-19 is not losing someone due to COVID-19, for change in main economic activity is not experiencing a change in main economic activity, and for change in living situation is not experiencing a change in living situation.

**
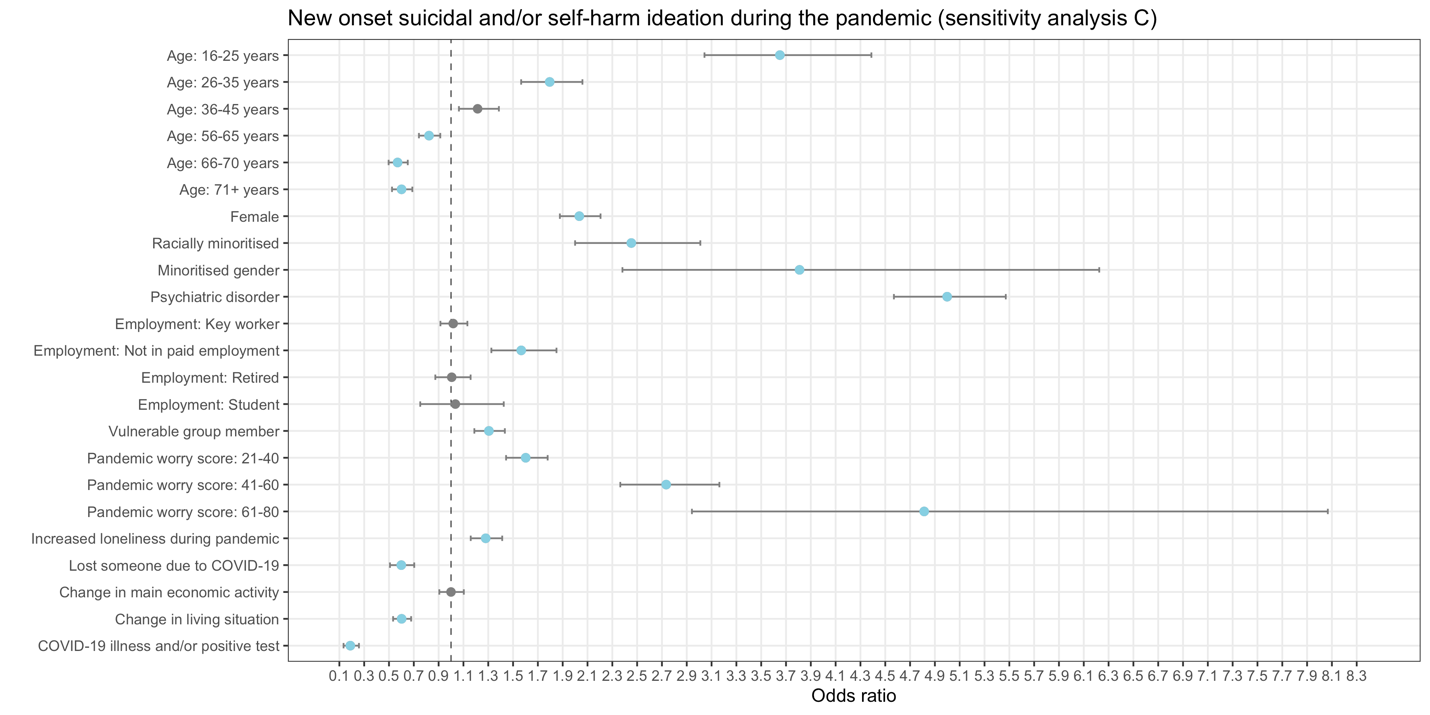
**

**SUPPLEMENTARY FIGURE 17** Association between demographic and COVID-related variables and new onset of suicidal and/or self-harm ideation during the pandemic. Results are derived from multiple logistic regression models in a sensitivity analysis in which participants must have answered ‘No’ at least three times to be considered to not have experienced the outcome during the monitoring period (*n* = 14,470). Points in blue are significant (*p* < 0.0038). We found a high correlation between the odds ratios from this analysis with the odds ratios from the main analysis (*r* = 0.96). *Note*. Minoritised gender = ‘Transgender’, ‘Non-binary’, and ‘Prefer to self-define’; Racially minoritised = ‘Arab’, ‘Asian’, ‘Black’, ‘Mixed race’, and ‘Other’. Reference category for age is 46-55 years, for female is being male, for racially minoritised is being white, for minoritised gender is not being minoritised gender, for psychiatric disorder is not having a psychiatric disorder, for employment is being in paid employment, for vulnerable group member is not being a vulnerable group member, for pandemic worry score is a score of 0-20, for losing someone due to COVID-19 is not losing someone due to COVID-19, for change in main economic activity is not experiencing a change in main economic activity, and for change in living situation is not experiencing a change in living situation.

**
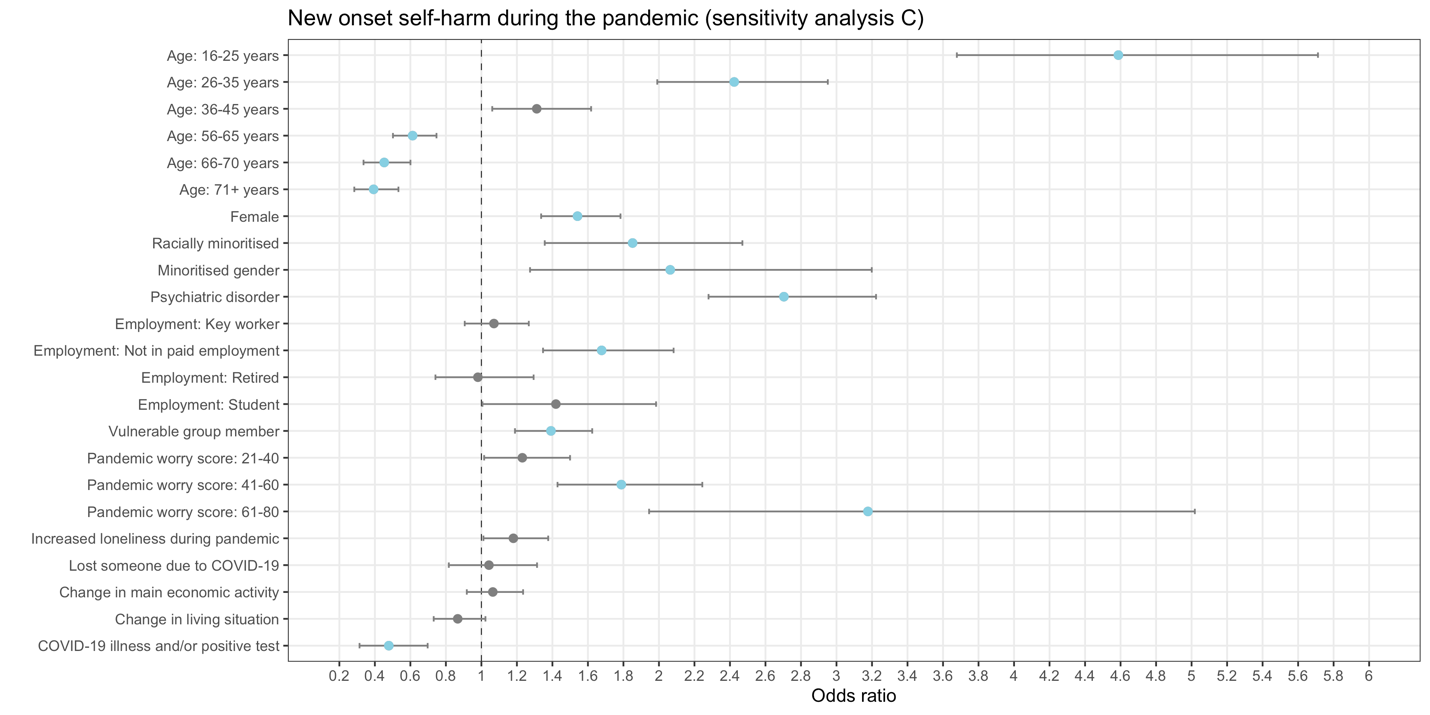
SUPPLEMENTARY FIGURE 18** Association between demographic and COVID-related variables and new onset of self-harm during the pandemic. Results are derived from multiple logistic regression models in a sensitivity analysis in which participants must have answered ‘No’ at least three times to be considered to not have experienced the outcome during the monitoring period (*n* = 22,849). Points in blue are significant (*p* < 0.0038). We found a high correlation between the odds ratios from this analysis with the odds ratios from the main analysis (*r* = 0.93). *Note*. Minoritised gender = ‘Transgender’, ‘Non-binary’, and ‘Prefer to self-define’; Racially minoritised = ‘Arab’, ‘Asian’, ‘Black’, ‘Mixed race’, and ‘Other’. Reference category for age is 46-55 years, for female is being male, for racially minoritised is being white, for minoritised gender is not being minoritised gender, for psychiatric disorder is not having a psychiatric disorder, for employment is being in paid employment, for vulnerable group member is not being a vulnerable group member, for pandemic worry score is a score of 0-20, for losing someone due to COVID-19 is not losing someone due to COVID-19, for change in main economic activity is not experiencing a change in main economic activity, and for change in living situation is not experiencing a change in living situation.

**
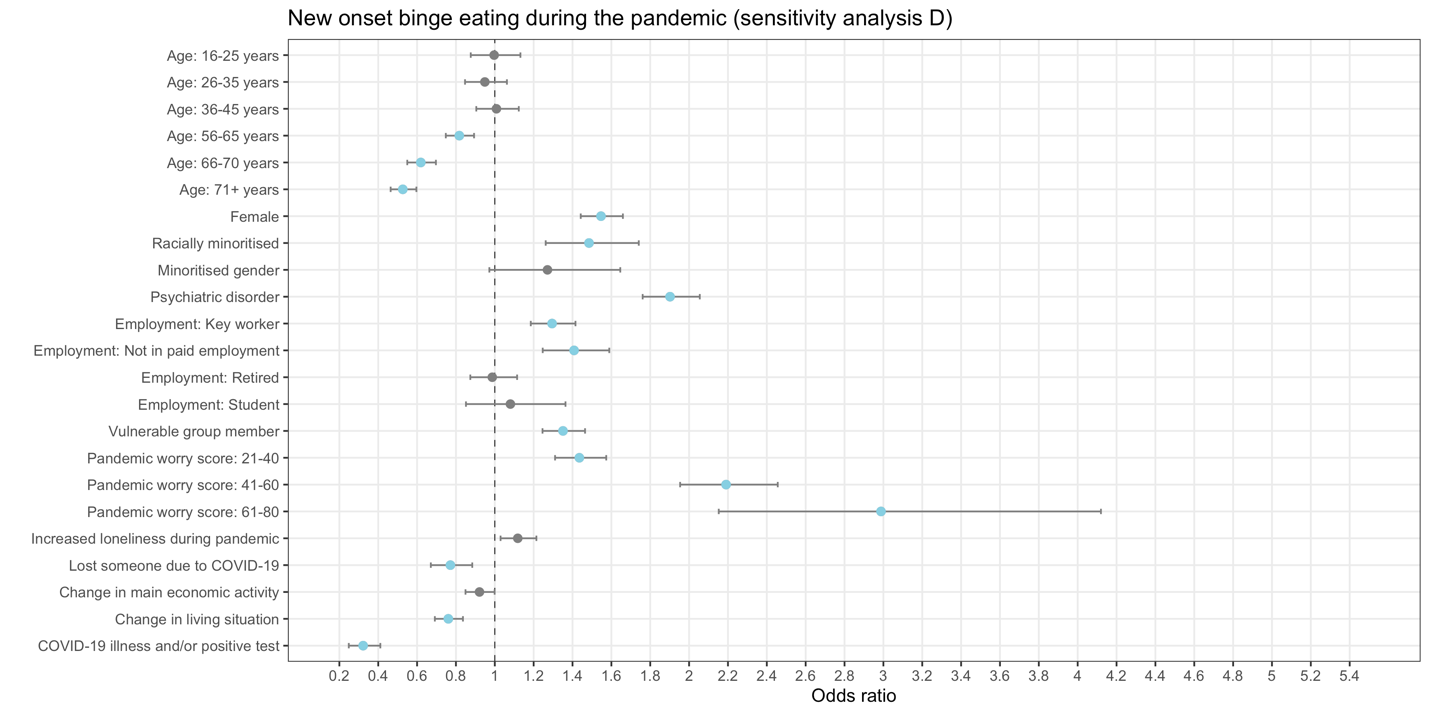
SUPPLEMENTARY FIGURE 19** Association between demographic and COVID-related variables and new onset of binge eating during the pandemic. Results are derived from multiple logistic regression models in a sensitivity analysis in which we only included instances in which the outcome was first reported in a phase after the exposure was first reported (*n* = 23,901). Points in blue are significant (*p* < 0.0038). We found a high correlation between the odds ratios from this analysis with the odds ratios from the main analysis (*r* = 0.99). *Note*. Minoritised gender = ‘Transgender’, ‘Non-binary’, and ‘Prefer to self-define’; Racially minoritised = ‘Arab’, ‘Asian’, ‘Black’, ‘Mixed race’, and ‘Other’. The results for age, being female, racially minoritised, and minoritised gender are the same as in the main analysis because the direction of causality cannot work in the opposite direction, thus it was appropriate to include cases in which the outcome was reported in the same phase as the exposure. The correlation is calculated between the odds ratios of all the remaining variables. Reference category for age is 46-55 years, for female is being male, for racially minoritised is being white, for minoritised gender is not being minoritised gender, for psychiatric disorder is not having a psychiatric disorder, for employment is being in paid employment, for vulnerable group member is not being a vulnerable group member, for pandemic worry score is a score of 0-20, for losing someone due to COVID-19 is not losing someone due to COVID-19, for change in main economic activity is not experiencing a change in main economic activity, and for change in living situation is not experiencing a change in living situation.


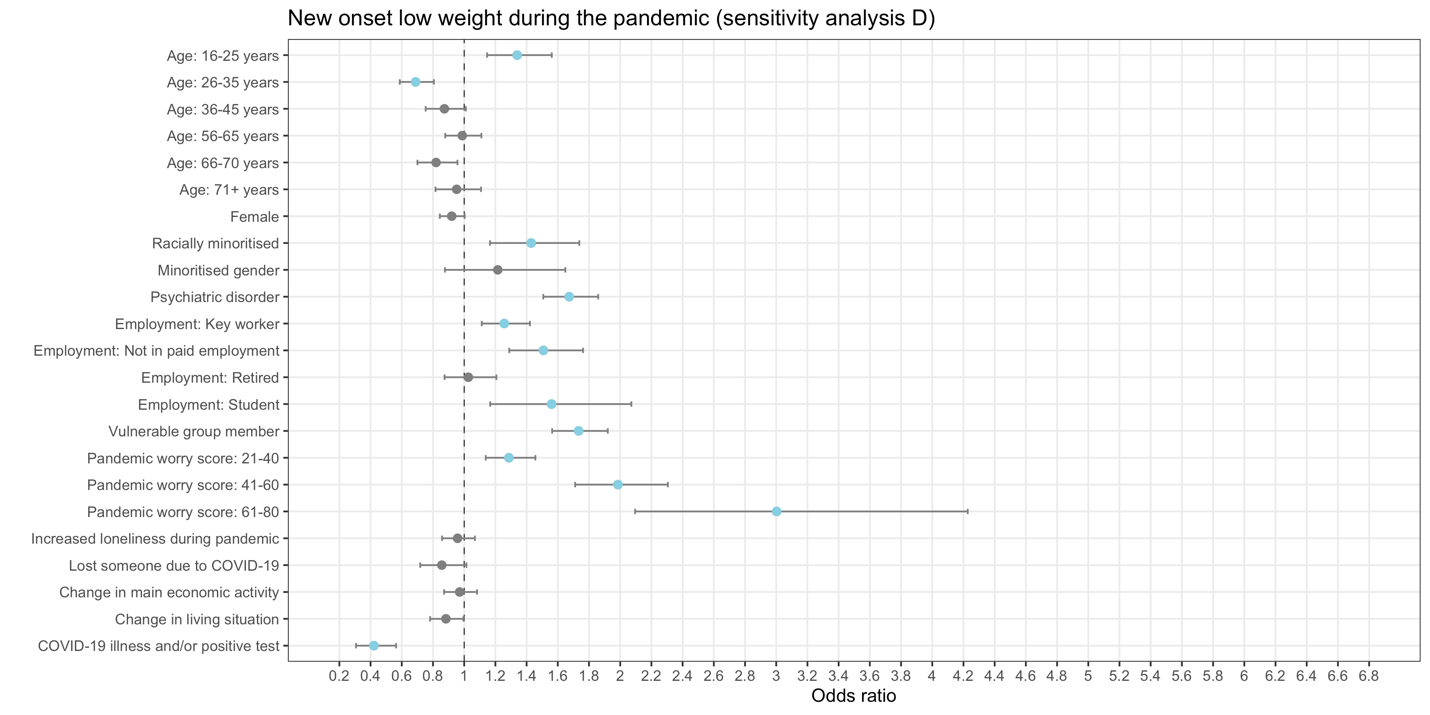


**SUPPLEMENTARY FIGURE 20** Association between demographic and COVID-related variables and new onset of low weight during the pandemic. Results are derived from multiple logistic regression models in a sensitivity analysis in which we only included instances in which the outcome was first reported in a phase after the exposure was first reported (*n* = 24,222). Points in blue are significant (*p* < 0.0038). We found a high correlation between the odds ratios from this analysis with the odds ratios from the main analysis (*r* = 0.99). *Note*. Minoritised gender = ‘Transgender’, ‘Non-binary’, and ‘Prefer to self-define’; Racially minoritised = ‘Arab’, ‘Asian’, ‘Black’, ‘Mixed race’, and ‘Other’. The results for age, being female, racially minoritised, and minoritised gender are the same as in the main analysis because the direction of causality cannot work in the opposite direction, thus it was appropriate to include cases in which the outcome was reported in the same phase as the exposure. The correlation is calculated between the odds ratios of all the remaining variables. Reference category for age is 46-55 years, for female is being male, for racially minoritised is being white, for minoritised gender is not being minoritised gender, for psychiatric disorder is not having a psychiatric disorder, for employment is being in paid employment, for vulnerable group member is not being a vulnerable group member, for pandemic worry score is a score of 0-20, for losing someone due to COVID-19 is not losing someone due to COVID-19, for change in main economic activity is not experiencing a change in main economic activity, and for change in living situation is not experiencing a change in living situation.

**
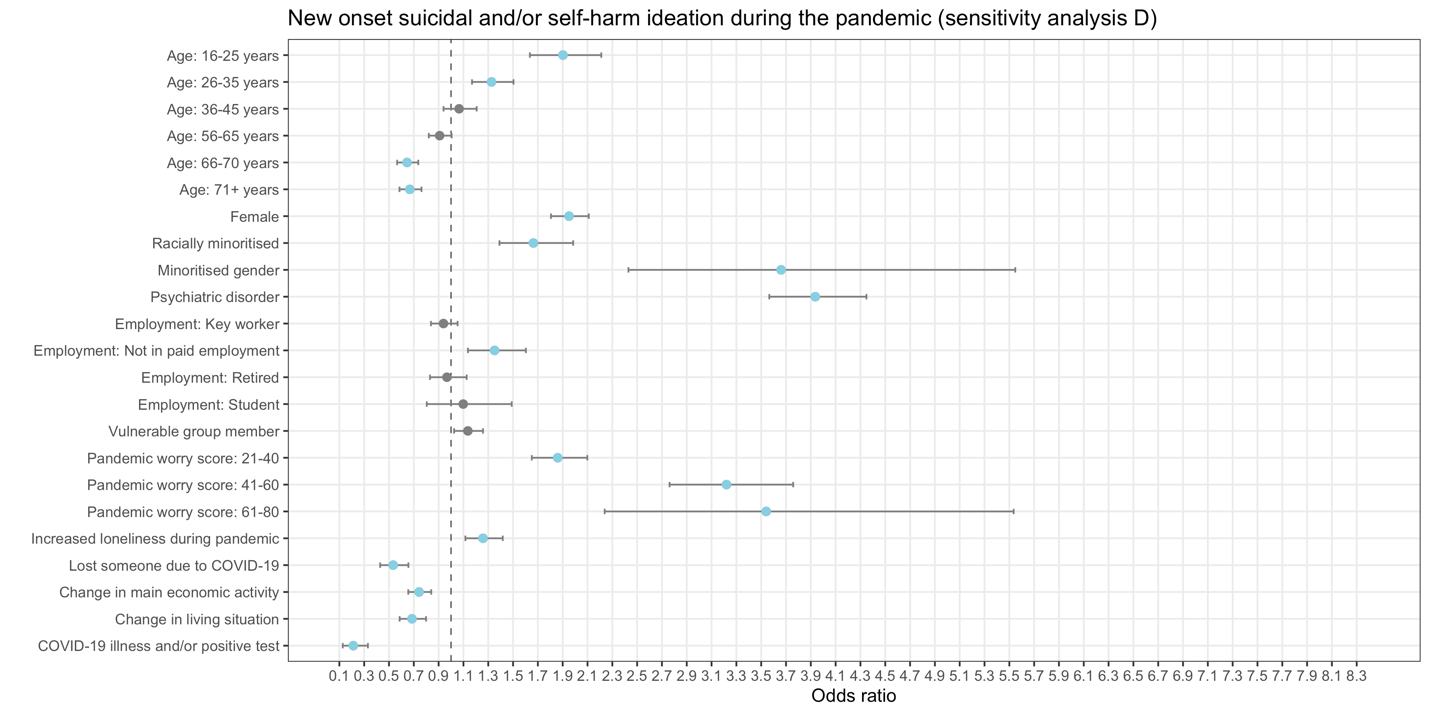
**

**SUPPLEMENTARY FIGURE 21** Association between demographic and COVID-related variables and new onset of suicidal and/or self-harm ideation during the pandemic. Results are derived from multiple logistic regression models in a sensitivity analysis in which we only included instances in which the outcome was first reported in a phase after the exposure was first reported (*n* = 17,433). Points in blue are significant (*p* < 0.0038). We found a high correlation between the odds ratios from this analysis with the odds ratios from the main analysis (*r* = 0.97). *Note*. Minoritised gender = ‘Transgender’, ‘Non-binary’, and ‘Prefer to self-define’; Racially minoritised = ‘Arab’, ‘Asian’, ‘Black’, ‘Mixed race’, and ‘Other’. The results for age, being female, racially minoritised, and minoritised gender are the same as in the main analysis because the direction of causality cannot work in the opposite direction, thus it was appropriate to include cases in which the outcome was reported in the same phase as the exposure. The correlation is calculated between the odds ratios of all the remaining variables.

Reference category for age is 46-55 years, for female is being male, for racially minoritised is being white, for minoritised gender is not being minoritised gender, for psychiatric disorder is not having a psychiatric disorder, for employment is being in paid employment, for vulnerable group member is not being a vulnerable group member, for pandemic worry score is a score of 0-20, for losing someone due to COVID-19 is not losing someone due to COVID-19, for change in main economic activity is not experiencing a change in main economic activity, and for change in living situation is not experiencing a change in living situation.

**
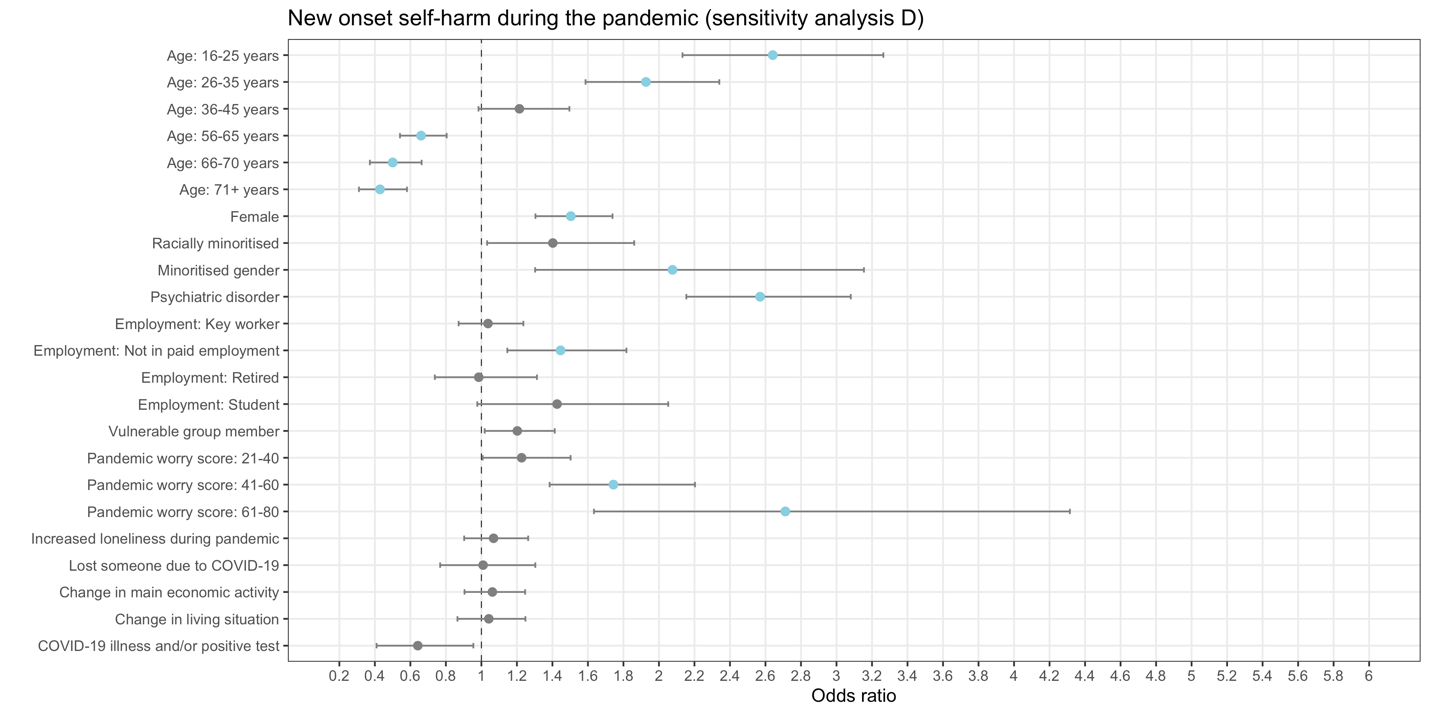
SUPPLEMENTARY FIGURE 22** Association between demographic and COVID-related variables and new onset of self-harm during the pandemic. Results are derived from multiple logistic regression models in a sensitivity analysis in which we only included instances in which the outcome was first reported in a phase after the exposure was first reported (*n* = 29,867). Points in blue are significant (*p* < 0.0038). We found a high correlation between the odds ratios from this analysis with the odds ratios from the main analysis (*r* = 0.99). *Note*. Minoritised gender = ‘Transgender’, ‘Non-binary’, and ‘Prefer to self-define’; Racially minoritised = ‘Arab’, ‘Asian’, ‘Black’, ‘Mixed race’, and ‘Other’. The results for age, being female, racially minoritised, and minoritised gender are the same as in the main analysis because the direction of causality cannot work in the opposite direction, thus it was appropriate to include cases in which the outcome was reported in the same phase as the exposure. The correlation is calculated between the odds ratios of all the remaining variables. Reference category for age is 46-55 years, for female is being male, for racially minoritised is being white, for minoritised gender is not being minoritised gender, for psychiatric disorder is not having a psychiatric disorder, for employment is being in paid employment, for vulnerable group member is not being a vulnerable group member, for pandemic worry score is a score of 0-20, for losing someone due to COVID-19 is not losing someone due to COVID-19, for change in main economic activity is not experiencing a change in main economic activity, and for change in living situation is not experiencing a change in living situation.

**References**

1. Davis, K. A. S., Coleman, J. R. I., Adams, M. & Allen, N. Mental health in UK Biobank–development, implementation and results from an online questionnaire completed by 157 366 participants: a reanalysis. *BJPsych* **6(20),** (2020).

2. Thornton, L. M. *et al.* The Anorexia Nervosa Genetics Initiative (ANGI): Overview and methods. *Contemp. Clin. Trials* **74**, 61–69 (2018).

3. Luce, K. H. & Crowther, J. H. The reliability of the eating disorder examination—Self-report questionnaire version (EDE-Q). *Int. J. Eat. Disord.* **25**, 349–351 (1999).
